# Multiparameter toxicity assessment of novel DOPO-derived organophosphorus flame retardants

**DOI:** 10.1007/s00204-016-1680-4

**Published:** 2016-02-29

**Authors:** Cordula Hirsch, Britta Striegl, Stephanie Mathes, Christian Adlhart, Michael Edelmann, Epifania Bono, Sabyasachi Gaan, Khalifah A. Salmeia, Lisa Hoelting, Alice Krebs, Johanna Nyffeler, Regina Pape, Alexander Bürkle, Marcel Leist, Peter Wick, Stefan Schildknecht

**Affiliations:** 1 Particles-Biology Interactions Laboratory, Swiss Federal Laboratories for Materials Science and Technology (Empa), Lerchenfeldstrasse 5, 9014 St. Gallen, Switzerland; 2 University of Konstanz, Universitaetsstr. 10, 78457 Konstanz, Germany; 3 ZHAW, Life Sciences and Facility Management, Einsiedlerstr. 31, 8820 Waedenswil, Switzerland; 4 Additives and Chemistry Group, Advanced Fibers, Swiss Federal Laboratories for Materials Science and Technology (Empa), Lerchenfeldstrasse 5, 9014 St. Gallen, Switzerland

**Keywords:** Flame retardants, Organophosphate, Dermal absorption, Neurotoxicity, Polybrominated diphenyls

## Abstract

Halogen-free organophosphorus flame retardants are considered as replacements for the phased-out class of polybrominated diphenyl ethers (PBDEs). However, toxicological information on new flame retardants is still limited. Based on their excellent flame retardation potential, we have selected three novel 9,10-dihydro-9-oxa-10-phosphaphenanthrene-10-oxide (DOPO) derivatives and assessed their toxicological profile using a battery of in vitro test systems in order to provide toxicological information before their large-scale production and use. PBDE-99, applied as a reference compound, exhibited distinct neuro-selective cytotoxicity at concentrations ≥10 µM. 6-(2-((6-oxido-6*H*-dibenzo[*c,e*][1,2]oxaphosphinin-6-yl)amino)ethoxy)-6*H*-dibenzo[*c,e*][1,2]oxaphosphinine 6-oxide (ETA-DOPO) and 6,6′-(ethane-1,2-diylbis(oxy))bis(6*H*-dibenzo[*c,e*][1,2]oxaphosphinine-6-oxide) (EG-DOPO) displayed adverse effects at concentrations >10 µM in test systems reflecting the properties of human central and peripheral nervous system neurons, as well as in a set of non-neuronal cell types. DOPO and its derivative 6,6′-(ethane-1,2-diylbis(azanediyl))bis(6*H*-dibenzo[*c,e*][1,2]oxaphosphinine-6-oxide) (EDA-DOPO) were neither neurotoxic, nor did they exhibit an influence on neural crest cell migration, or on the integrity of human skin equivalents. The two compounds furthermore displayed no inflammatory activation potential, nor did they affect algae growth or daphnia viability at concentrations ≤400 µM. Based on the superior flame retardation properties, biophysical features suited for use in polyurethane foams, and low cytotoxicity of EDA-DOPO, our results suggest that it is a candidate for the replacement of currently applied flame retardants.

**Electronic supplementary material:**

The online version of this article (doi:10.1007/s00204-016-1680-4) contains supplementary material, which is available to authorized users.

## Introduction

Flame retardants are added to a wide range of materials in order to lower the risk of uncontrolled ignition and reduce combustion rates. In recent years, the class of polybrominated diethyl ether retardants has been phased out based on a growing understanding of their abundant prevalence in the environment and their persistence and bioaccumulation in the food chain (Covaci et al. 2011; Shaw et al. 2010; Hites 2004). Several reports have indicated adverse effects of polybrominated flame retardants on endocrine functions mediated, for example, by thyroid-, androgen-, and estrogen-signaling pathways (Zhou et al. 2002; Li et al. 2013; Stoker et al. 2005), but also on kidney and liver integrity (Albina et al. 2010), the cholinergic system (Viberg et al. 2002), neuronal differentiation (Dingemans et al. 2007; Herbstman et al. 2010), and migration of neuronal precursors during development (Schreiber et al. 2010). Strict legal requirements regarding flammability properties are only one issue restricting the delicate choice of an alternative to brominated flame retardants. In addition, to legal issues, toxicological information plays an important role in protecting human safety by ensuring informed use of potentially harmful agents; however, such information is limited, while legal requirements for industry to disclose the chemical nature and toxicological properties of manufactured compounds are insufficient.

As a promising approach for the replacement of polybrominated diphenyl ethers that represent a major environmental burden (Wu et al. 2012), phosphorus flame retardants such as inorganic red phosphorus or ammonium polyphosphate, halogenated phosphorus flame retardants, phosphonamidates, and phosphoesters (Costa and Giordano 2007; van der Veen and de Boer 2012) are currently applied to meet regulatory flammability standards. Several of these replacements are additives and not covalently bound to their host material, thus allowing their diffusion out of the host material by volatilization, leaching, or mechanical abrasion (Marklund et al. 2003). In case of the chlorinated phosphorus flame retardant tris(chloropropyl)phosphate (TCPP), which was intensively applied as an alternative to brominated additives, concentrations in the environment, indoor ambient air, and household dust exceed those of polybrominated diphenyl ethers (PBDEs; Staaf and Ostman 2005; Tollbäck et al. 2006; van der Veen and de Boer 2012; Martínez-Carballo et al. 2007). Recent observations have indicated the carcinogenic potential of TCPP (Ni et al. 2007; WHO 1998), justifying concerns regarding whether the alternatives currently used actually represent a lower risk than that of the compounds they are supposed to replace (Behl et al. 2015). These safety concerns regarding the industrial application of phosphorus flame retardants deserve considerable attention because structurally related phosphodiester compounds, including organophosphorus pesticides, adversely influence the development of the nervous system and are used as chemical warfare agents (González-Alzaga et al. 2014; Muñoz-Quezada et al. 2013).

One of the potential substituents, which represents the basis for the here introduced novel structures, is the halogen-free organophosphorus flame retardant 9,10-dihydro-9-oxa-10-phosphaphenanthrene-10-oxide (DOPO), which displays excellent flame retardation properties and exhibited low acute toxicity potential in currently available studies (Waaijers et al. 2013a, b). In order to improve its flame retardation potential and influence its physico-chemical parameters, such as its melting point, to allow industrial application in different plastic materials, novel phosphonamidate and phosphonate and phosphinate derivatives of DOPO have been developed (Gaan et al. 2009, 2013, 2015; Buczko et al. 2014; Neisius et al. 2014; Salmeia and Gaan 2015; Salmeia et al. 2015), but have not been evaluated with respect to their toxicological potential. In the combustion process, phosphonamidate DOPO derivatives primarily act in the vapor phase by forming phosphorus-containing radical species (PO^·^) (Salmeia and Gaan 2015; Salmeia et al. 2015), which interact with ^·^OH or H^·^ radicals (Eq. 1) formed during combustion and therefore lead to a reduction in the release of heat, favoring the extinguishing of the flame. In a subsequent step, the reduced phosphorus compound interacts with a second H^·^ radical (or ^·^OH), allowing recycling of the PO^·^ species (Eq. 2).

1 $${\text{PO}}^{ \cdot } + {\text{H}}^{ \cdot } \to {\text{HPO}}$$

2 $$2{\text{HPO}} + 2{\text{H}}^{ \cdot } + {\text{O}}_{2} \to 2{\text{PO}}^{ \cdot } + 2{\text{H}}_{2} {\text{O}}$$

The application of such novel DOPO-derived phosphorus flame retardants is currently precluded by limited information regarding their adverse effects on human development and health. In the present study, we assessed three novel *bis*-DOPO-derived flame retardants in direct comparison with their parental compound DOPO and polybrominated diphenyl ether PBDE-99 with respect to their acute cytotoxic potential, their interaction with epidermal barriers, and their influence on neuronal systems. Epidermal uptake was investigated by confocal Raman spectroscopy in ex vivo skin samples, the influence of the flame retardants on epidermal architecture was investigated in a 3D human epidermal in vitro model, and the irritative potential of the compounds on the cellular level was analyzed in a keratinocyte assay allowing the quantitative detection of electrophilic skin-sensitizing compounds. To analyze the influence of the flame retardants tested herein on the nervous system, in vitro models of central and peripheral neurons were utilized. Neurons were exposed to flame retardants either in a fully mature state (indicator for neurotoxicity) or during the differentiation process (indicator for developmental neurotoxicity). A potential manipulation of early-stage developmental processes was finally tested in a human in vitro neural crest cell model by which the influence on cell migration was assessed as readout. These toxicological investigations, together with a comparison of flame retardation properties when the compounds were applied as additives in polyurethane foams, indicated that 6,6′-(ethane-1,2-diylbis(azanediyl))bis(6*H*-dibenzo[*c,e*][1,2]oxaphosphine-6-oxide) (EDA-DOPO) combines superior flame retardation with foaming process compatibility in comparison with that of DOPO; therefore, EDA-DOPO might emerge as a new substitute for currently applied flame retardants.

## Materials and methods

### UL94-HB (horizontal burning) fire tests

The UL-94 (ATSM D 4986 or ISO 9772) flammability standard test was applied to classify the flame retardation potential of the organophosphorus compounds embedded as additives in polyurethane (PU). The PU foam was cut into pieces measuring 150 mm × 50 mm × 13 mm (length × width × thickness). Test specimens were marked at 25, 60, and 125 mm. The test specimens were placed on an iron grid in a horizontal position above a cotton indicator. A 38-mm flame was then applied to the end of the specimen for 60 s. The time required to burn a 100-mm-long segment was recorded. The flame retardation potential of the test compounds was classified according to the UL-94 horizontal flammability criteria: Classification HF 1: ≤2 s burning time after removal of the external flame in 80 % of the test specimens and ≤10 s in the remaining 20 % of test specimens, ≤30 s afterglow time for each individual specimen, <60 mm damaged length for each specimen, and no ignition of the cotton indicator by burning drops or particles. Classification HF 2: same criteria as HF 1 with the exception that the cotton is ignited by burring drops or particles. Classification HBF: burning rate ≤40 mm/min over a distance of 100 mm or burning length ≤125 mm. The UL 94 horizontal rating classifications are ranked as follows: HF1 > HF2 > HBF > no rating.

### Toxicity assessment in human alveolar epithelial cells

The human alveolar epithelial cell line A549 (ATCC: CCL-185) was grown in Roswell Park Memorial Institute (RPMI-1640) medium (Sigma-Aldrich) supplemented with 10 % FCS (Lonza), 2 mM l-glutamine (Gibco), 50 µg/mL penicillin (Gibco), 50 µg/mL streptomycin (Gibco), and 100 µg/mL neomycin (Gibco) at 37 °C in a humidified atmosphere containing 5 % CO_2_ (hereafter referred to as complete cell culture medium and standard growth conditions, respectively). Cells were subcultured at approximately 80–90 % confluency using 0.5 % trypsin–EDTA (Sigma-Aldrich). For viability assessment, 1.5 × 10^4^ cells per well were seeded in 200 µL complete cell culture medium in a 96-well plate and grown overnight under standard growth conditions, after which the cells were treated for 48 h with 200 µL standard growth medium per well containing increasing concentrations of the flame retardants or positive control agent CdSO_4_. CellTiter96^®^ AQueous One Solution (Promega) containing MTS (3-(4,5-dimethylthiazol-2-yl)-5-(3-carboxymethoxy-phenyl)-2-(4-sulfophenyl)-2*H*) as a water-soluble tetrazolium compound was used according to the manufacturer’s protocol. Absorption was detected at 490 nm.

### Toxicity assessment in human macrophages

The human monocytic cell line THP-1 (ATCC: TIB-202) was grown as cell suspension in complete cell culture medium under standard growth conditions. Subculturing was performed by replacement of the medium when the cell density reached 8 × 10^5^ cells/mL. Cell concentrations were not allowed to exceed 10^6^ cells/mL. To differentiate THP-1 monocytes into macrophages, cells were grown in the presence of 200 nM phorbol-12-myristate-13-acetate (PMA; Sigma-Aldrich) for 72 h. For viability assessment, 8 × 10^4^ cells per well were seeded in 200 µL complete cell culture medium containing 200 nM PMA in a 96-well plate and differentiated for 72 h under standard growth conditions. Thereafter, the medium was removed, the cells were washed twice with pre-warmed phosphate-buffered saline (PBS), and increasing concentrations of flame retardants or CdSO_4_ were added in 200 µL complete cell culture medium per well for 48 h. CellTiter96^®^ AQueous One Solution (Promega, Switzerland) containing MTS (3-(4,5-dimethylthiazol-2-yl)-5-(3-carboxymethoxy-phenyl)-2-(4-sulfophenyl)-2*H*) as a water-soluble tetrazolium compound was used according to the manufacturer’s protocol. Absorption was detected at 490 nm.

## 3D epidermal in vitro model

Human primary keratinocytes (PR3D-HPEK-50, CELLnTEC, Switzerland) were grown in flasks in proliferation medium (CNT-PR, CELLnTEC) until subconfluency was reached, after which the cells were seeded into transwell inserts containing polycarbonate membranes with a pore size of 0.4 µm at a density of 3.3 × 10^5^ cells/cm^2^ and grown for 3 days in CNT-PR until confluency was reached. Differentiation was initiated by the addition of 3D barrier medium (CNT-PR-3D, CELLnTEC) for 18 h to allow development of cell-to-cell adhesion structures. 3D growth and differentiation were induced by complete removal of medium from the upper transwell compartment and replacement of the CNT-PR-3D differentiation medium in the lower compartment. Cells were allowed to differentiate for 11 days with 3 medium changes per week in the lower compartment and direct cell-to-air contact in the top compartment. Flame retardants were suspended at a concentration of 200 mM in PBS containing 5 % DMSO, after which 100 µL of each test compound was applied on top of the epithelial barrier model. Five percent SDS (sodium dodecyl sulfate) was used as a cytotoxic positive control compound, whereas 5 % DMSO in PBS was used as a negative control compound. The epidermal models were treated for 15 min at 37 °C and washed 3 times with PBS, after which they were further cultivated for 48 h in fresh medium. Cell viability of the epidermal models was assessed by measuring the reduction of resazurin to resorufin (PrestoBlue^®^ Assay). Cell viability values (fluorescence signals) were normalized to the epidermal model thickness. Tissues were later washed with PBS and fixed in 10 % formalin at room temperature for 2.5 h. The membranes carrying the 3D epidermis were removed from the transwell plates and washed using PBS. The 3D epidermis was dehydrated with the consecutive addition of: 70 % EtOH for 1 h; 96 % EtOH for 1 h; 96 % EtOH for 1 h, 100 % EtOH for 1 h, 100 % EtOH for 1 h; extraction solvent for 1 h; extraction solvent for 1.5 h, paraffin for 1.5 h, paraffin for 1.5 h. Paraffin blocks were cut with a microtome (Zeiss, Hyrax M40) into 5-µm slices and heated at 55 °C for 1 h. Samples were re-hydrated in xylene and decreasing ethanol concentrations (2 min) and placed in water for 2 min, after which they were stained with hematoxylin for 15 s. After hematoxylin staining, the slides were “blued” by rinsing them under tap water (5 min) and exposing them to 75 % EtOH and 96 % EtOH for 2 min, after which they were stained with alcoholic eosin (4 min). Finally, the slides were washed with 96 % EtOH and dehydrated with 100 % EtOH and xylene. Samples were mounted with DPX mounting medium (Sigma-Aldrich) and analyzed by microscopy (Olympus IX 81).

### Skin sensitization assay

Generation of the stable KeratinoSens™ cell line was previously described in detail (Emter et al. 2010). KeratinoSens™ cells were maintained in DMEM containing glutamax (Gibco/Invitrogen), 9 % FCS, and 0.5 mg/mL G418. For the experiments, KeratinoSens™ cells were seeded onto 96-well plates at a density of 10,000 cells per well in 125 µL medium without G418. After 24 h, the medium was replaced with 200 µL DMEM containing 1 % FCS and the test compounds. After a 2-day incubation period at 37 °C in an atmosphere containing 5 % CO_2_, the cells were washed once with PBS and a resazurin reduction assay (PrestoBlue^®^) was carried out in order to compare the viability of the differently treated groups. Fluorescence intensity was measured at 550 nm_ex_ and 590 nm_em_. After another washing step with PBS, cell lysis was performed using 20 µL/well passive lysis buffer (Promega) for 20 min at room temperature. Luciferase activity was measured by applying 50 µL luciferase substrate automatically to each well and detecting the resulting luminescence. Compounds were considered as skin sensitizers when a statistically significant induction of luciferase activity above a defined threshold of ≥1.5 in the absence of impaired cell viability was observed (Emter et al. 2010; Natsch et al. 2011).

### Quantitative detection of flame retardants in porcine skin by confocal Raman spectroscopy

The vertical distribution of topically applied DOPO derivatives and reference compounds was investigated on segments of porcine ears from freshly slaughtered animals. The skin was separated from the cartilage, depilated, and placed into Franz diffusion cells (1 cm^2^). The acceptor solution was PBS buffer containing 2 % IGEPAL^®^ CA-630 (Sigma-Aldrich). The test compounds were dispersed at a concentration of 400 µM (2 % in the case of caffeine) in PBS buffer containing 2 % IGEPAL^®^ CA-630, after which 300 µL of this mixture (or a blank solution) was topically applied onto the porcine skin and allowed to penetrate for 60 min. Next, the skin was removed from the Franz cells, gently washed with PBS, and placed onto the optical window of an inverse confocal Raman microscope (Model 3510 SCA, *River Diagnostics)* equipped with a 60 × oil-immersion objective. The 785-nm excitation laser was used to acquire spectra in the fingerprint region (400–1800 cm^−1^, 5 s integration time) starting from the surface of the skin and extending to a depth of 60 µm (2 µm step size). The acquisition was repeated for at least five positions. The averaged concentration profiles of the flame retardants and reference compounds were extracted by fitting (unrestricted classical least squares, third-order polynomial baseline) the Raman spectra with the reference spectra of keratin (as representative of all proteins), water, ceramide 3, cholesterol (as representatives of all lipids), natural moisturizing factor (NMF; fixed composition), lactate, urea, and solid test compounds using Skin Tools 2.0 software (River Diagnostics). The concentrations of the test substances are given as the molar fraction relative to keratin rather than correcting for the Raman signal attenuation using mathematical models (Franzen et al. 2013). The response factors of the test compounds were determined against BSA solutions assuming similar Raman cross sections for keratin and BSA as described in the literature (Caspers et al. 2001; Fleischli et al. 2013, 2015). Because EDA-DOPO, ETA-DOPO, and EG-DOPO were only partially soluble in chloroform, their spectra were calibrated against DOPO with the assumption of the same Raman cross section for the biphenyl group. Because of the nature of the unrestricted fit, negative fitting coefficients can be obtained and the values must be compared to those of the blank sample. The fitting coefficients of the blank, untreated skin were also used to estimate the level of detection. The depth resolution of the confocal microscope was determined to be 8 µm at FWHM, leaving an uncertainty of approximately 4 µm in differentiating between truly permeated compounds and compounds sitting at the surface of the skin.

### Toxicity assessment of central nervous system neurons

LUHMES cells are conditionally immortalized human fetal ventral mesencephalic neuronal precursor cells that were obtained by clonal selection. Differentiated LUHMES cells show a clear dopaminergic phenotype, which was described in detail previously (Schildknecht et al. 2009; Scholz et al. 2011). Cells were propagated in advanced DMEM/F12 (Gibco/Invitrogen, Darmstadt, Germany) with 1 × N2 supplement (Invitrogen), 2 mM l-glutamine (Gibco), and 40 ng/mL recombinant bFGF (R&D Systems, Minneapolis, MN, USA). The differentiation process was initiated by adding differentiation medium consisting of advanced DMEM/F12, 1 × N2 supplement, 2 mM l-glutamine, 1 mM dibutyryl cAMP (Sigma), 1 µg/mL tetracycline (Sigma), and 2 ng/mL recombinant human GDNF (R + D Systems). After 2 days, cells were trypsinized, collected in advanced DMEM/F12 medium, and seeded onto 24-well plates at a density of 160,000 cells/cm^2^. The differentiation process was continued for 4 days. Fully differentiated LUHMES cells were treated with the tested flame retardants for 48 h from day 6 to 8 of differentiation. For visualization of morphology, cells were fixed with 4 % paraformaldehyde for 20 min at room temperature, permeabilized with 0.2 % Triton X-100, washed with PBS, blocked with 1 % BSA (Calbiochem, San Diego, CA, USA) in PBS for 1 h, and stained with a polyclonal anti-β-III-tubulin antibody (Sigma) in 1 % BSA/PBS at 4 °C overnight. After washing, secondary antibodies (anti-mouse-IgG, Alexa 488, Molecular Probes; 1:1000) in 1 % BSA/PBS were added for 1 h, after which nuclei were stained with Hoechst dye H-33342 (1 µg/mL) for 20 min. For visualization, an Olympus IX 81 microscope (Hamburg, Germany) equipped with an F-view CCD camera was used. For quantitative evaluation of the neurite area, β-III-tubulin-stained cells were analyzed using an automated microplate-reading microscope (Array-Scan II^®^ HCS Reader, Cellomics, Pittsburgh, PA, USA) equipped with a Hamamatsu ORCA-ER camera (1024 × 1024 resolution; run at 2 × 2 binning). Nuclei were identified according to their intensity, size, area, and shape. A virtual area corresponding to the cell soma was defined around each nucleus. The total β-III-tubulin pixel area per field minus the soma areas in that field was defined as neurite mass.

### Toxicity assessment of peripheral nervous system neurons

The human pluripotent stem cell (hPSC) line H9 (WA09 line) was obtained from WiCell (Madison, WI, USA). Import of cells and experiments was authorized under license #170-79-1-4-27 (Robert Koch Institute, Berlin, Germany). Cells were cultured according to standard protocols (Thomson et al. 1998) and differentiated into dorsal root ganglia-like cells as described earlier (Chambers et al. 2012), with slight modifications. Briefly, neural differentiation was promoted by adding neural differentiation medium and the combination of 6 small molecule pathway inhibitors [noggin (35 ng/mL; R&D Systems, Minneapolis, MN, USA), dorsomorphin (600 nM; Tocris Bioscience, Bristol, UK), SB-431642 (10 μM; Tocris Bioscience, Bristol, UK), CHIR99021 (1.5 µM; Axon Medchem, Vienna, VA, USA), SU5402 (1.5 µM; Tocris Bioscience, Bristol, UK), and DAPT (5 µM; Merck, Darmstadt, Germany)]. The cells were cryopreserved on day 8′ of differentiation (DoD 8′). For each experiment, cells were thawed in neural differentiation medium supplemented with CHIR99021 (1.5 µM), SU5402 (1.5 µM), and DAPT (5 µM). For the peripheral neurotoxicity test (PeriTox-test), test chemicals were serially diluted in differentiation medium containing BDNF, GDNF, and NGF (25 ng/mL; R&D Systems, Minneapolis, USA) and added to the cells on day 3 after thawing. After 48 h, neurite area and viability were assessed as described earlier (Krug et al. 2013). Briefly, cells were loaded with 1 µM calcein-AM and 1 µg/mL H-33342 for 1 h at 37 °C. For image acquisition, an Array-Scan VTI HCS (high content screening) microscope (Cellomics, Waltham, MA USA) was used. In an automated procedure, all H-33342^+^/calcein^+^ cells were analyzed as viable cells. In channel 1, H-33342^+^ nuclei were detected according to their size, area, shape, and intensity at excitation/emission wavelengths of 365 ± 50/535 ± 45. The nuclei borders were expanded radially by 3.2 µm to define a virtual cell soma area (VCSA). In channel 2 (at excitation/emission wavelengths of 474 ± 40/535 ± 45), all calcein^+^ pixels were identified as viable cell structures (VCS). An algorithm was applied that used the VCSA as a filter in the calcein channel and subtracted it from the VCS. The remaining pixels (VCS-VCSA) in the calcein channel were defined as neurite area and were represented relative to the untreated control.

### Neural crest cell (NCC) migration assay

Human embryogenic stem cells (H9 hESC line; Wisconsin International Stem Cell Bank, Madison, WI, USA) were plated on a confluent layer of mitomycin C-treated murine bone marrow-derived stromal MS5 cells and differentiated as previously described (Lee et al. 2010; Zimmer et al. 2012). After 21 days, rosettes were picked and plated onto poly-l-ornithine/laminin/fibronectin-coated plates in N2 medium. After 7 days, cells were FACS-sorted for positive expression of the neural crest marker human natural killer-1 (HNK-1) and expanded in N2 medium supplemented with epidermal growth factor (EGF, 20 ng/mL) and fibroblast growth factor (FGF2, 20 ng/mL). For expansion, the cells were passaged every 6 days using accutase (PAA, Pasching, Austria) to detach the cells. After 30 days of expansion, the cells were cryopreserved in 90 % fetal bovine serum, 10 % DMSO. For the analysis of neural crest cell migration, silicone stoppers (Platypus Technologies, Madison, WI, USA) were inserted in a 96-well plate coated with poly-l-ornithine/laminin/fibronectin. Freshly thawed NCCs were seeded at a density of 100,000 cells/cm^2^ in N2 medium containing EGF and FGF2 and allowed to attach for 1 day. Stoppers were removed, and the cells were allowed to migrate into the center of the well. Compounds tested in this study were diluted from a 10 mM stock in DMSO and were applied for 24 h. For single cell detection, cells were stained with H-33342 (1 µg/mL) and calcein-AM (0.5 µM) and imaged using a high content imaging microscope (Cellomics ArrayScanVTI, Thermo Fischer). Viability was assessed by counting the number of H-33342 and calcein double-positive cells outside the migration zone. Migration was detected by acquisition of the number of H-33342 and calcein double-positive cells in the migration area. Viability and migration were both normalized to untreated controls, and DMSO concentrations did not exceed 1 % in all experiments.

### Calculation of IC_50_ values

IC_50_ values are defined by the concentration of the flame retardant investigated where the response was reduced by half. Log-transformed flame retardant concentration values and the respective response data were fitted to a four-parameter logistic equation:

$$y = A + \frac{B - A}{{1 + (x/C)^{D} }}$$

The percent inhibition response data are represented by *y*; *B* = maximum of *y*; *A* = minimum of *y*; *x* = flame retardant concentration; *C* = IC_50_; *D* = Hill slope factor. The IC_50_ values were calculated from these sigmoidal dose–response curves utilizing Prism 5.0 (GraphPad, San Diego, CA, USA) software.

### Statistics

Values are expressed as mean ± SD. If not otherwise indicated, experiments were performed at least three times with three technical replicates in each experiment. Data were analyzed by one-way ANOVA or Student’s *t* test as appropriate. Following ANOVA, differences were determined by Bonferroni’s post hoc test (Prism or Origin software). If not otherwise indicated, differences between means were considered statistically significant at *p* < 0.05.

## Results

### Synthesis and flame retardation properties of DOPO derivatives

**Fig. 1:**
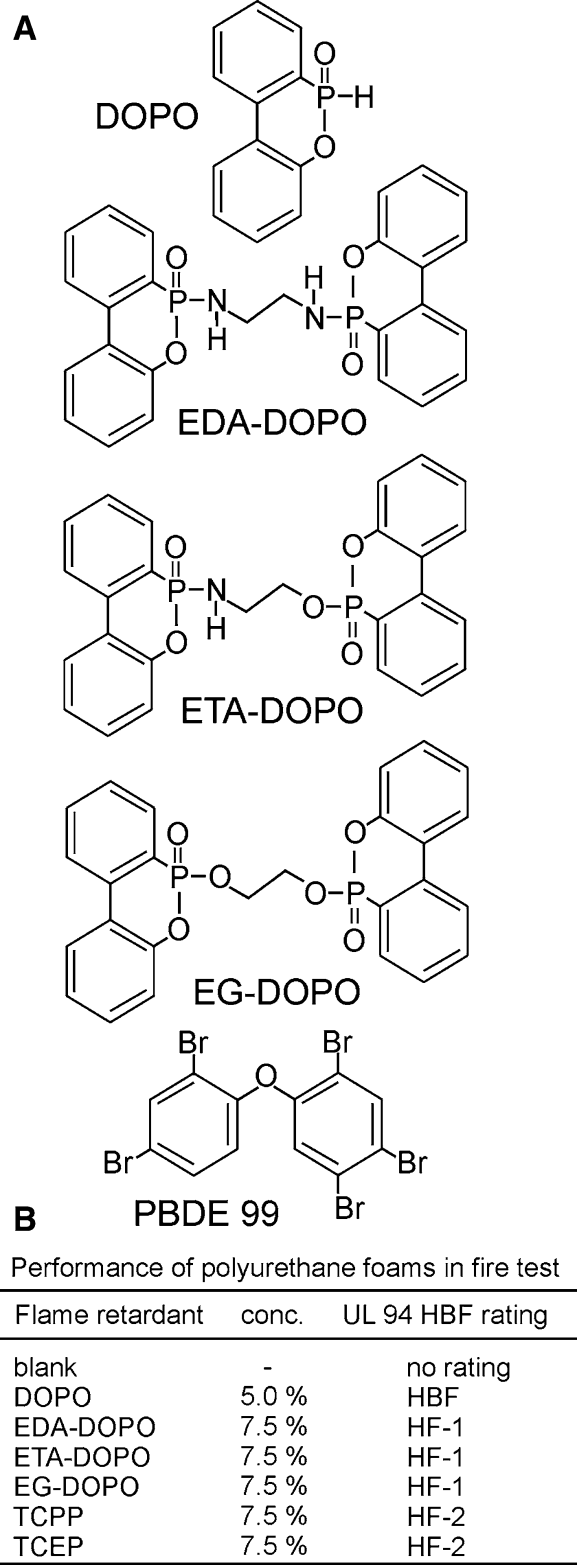
**a** Structures of 9,10-dihydro-9-oxa-10-phosphaphenanthrene-10-oxide (DOPO) and novel bridged DOPO derivatives EDA-DOPO, ETA-DOPO, and EG-DOPO. The derivatives are characterized by variations in the heteroatom attached to the phosphorus center of the DOPO monomer, comprising two phosphate moieties (EG-DOPO), two phosphoramidate moieties (EDA-DOPO), or a combination of phosphate and phosphoramidate moieties. **b** UL-94 horizontal burning fire test. The flame retardants were added to polyurethane foam at the indicated concentrations. The concentration in the host material was based on the weight of the polyol used in the manufacturing process of polyurethane foam. The density of the polyurethane foam was approximately 50 kg/m^3^. Based on the inherent reactivity of DOPO, its final concentration in the foam was limited to 5 %. UL-94 horizontal rating classification: HF 1 > HF 2 > HBF > no rating. TCPP (tris(2-chloroisopropyl)phosphate) and TCEP (tris-(2-chloroethyl)phosphate) were included in the fire tests as representative examples of widely applied chlorophosphates

In order to modulate the biophysical properties of DOPO to optimize it for use in polyurethane foams and further improve its flame retardation potential, three novel *bis*-DOPO derivatives comprising an alkyl spacer linked via N-and O-atoms to the central phosphate moieties were synthesized (Fig. 1a; Kobayashi et al. 2013; Gaan et al. 2013). The flame retardation potential was evaluated by subjecting the new compounds, embedded into polyurethane, to the UL-94 horizontal burning fire test (Fig. 1b). When added to soft polyurethane foams, the three novel bridged DOPO derivatives achieved flame retardation ratings higher than those of chlorophosphates TCCP and TCEP, which have been applied widely as reference compounds (Fig. 1b). The flame retardation potential of the DOPO derivatives was significantly higher than that of their parental compound DOPO (UL 94 horizontal rating classification: HF 1 > HF 2 > HBF; Fig. 1b). The fire burning test clearly indicated that the novel *bis*-DOPO derivatives display superior flame retardation properties in comparison with those of the compounds they are intended to replace. These features confirm that the novel *bis*-DOPO derivatives are new candidates for potential use in several plastic materials comprising everyday common household goods, while raising the question of their adverse influence on human health.

### Comparison with existing literature data

**Fig. 2:**
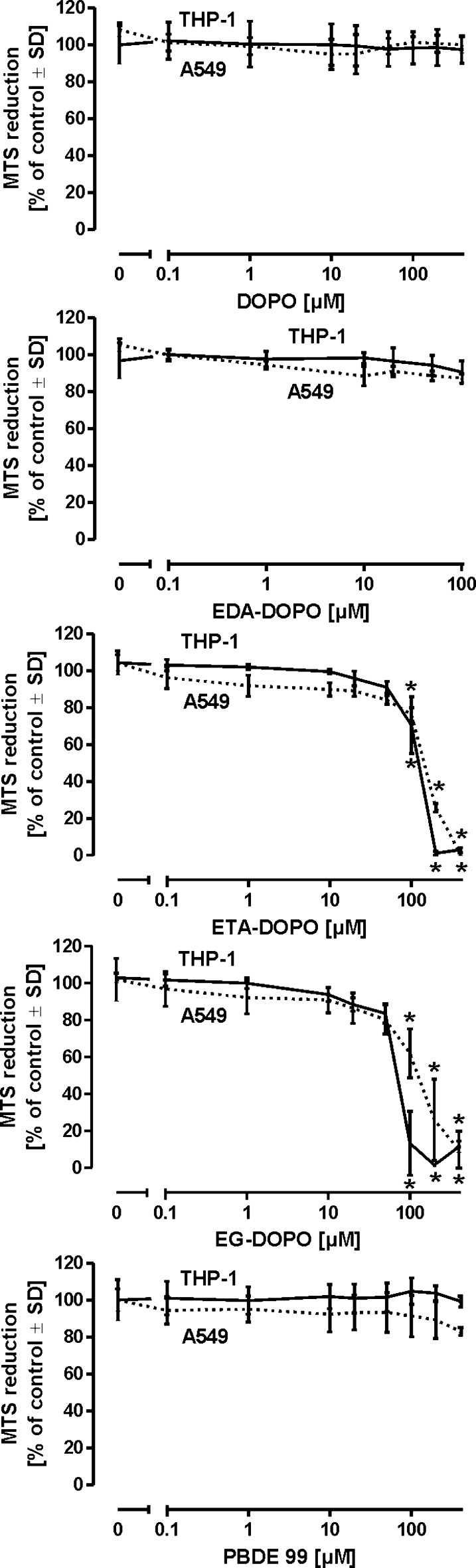
Determination of acute cytotoxicity. Human alveolar epithelial cells (A549) and human monocytes (THP-1) differentiated into macrophages were treated with the flame retardants for 48 h. Viability was assessed by measuring reduction of the tetrazolium compound MTS. All data are mean ± SD (*n* = 3); *p < 0.05

Because of their superior flame retardation potential and suitability for application in polyurethane foams, the three novel DOPO derivatives were tested in a series of in vitro models in order to obtain initial information on their potential harmful properties. To allow comparison with existing toxicological data for other flame retardants, DOPO and PBDE-99, as a representative polybrominated diethyl ether, were tested in parallel in all assays applied in this work. Inhalation is considered to be a primary route of exposure for flame retardants. Therefore, we first exposed A549 human lung epithelial cells and human macrophages differentiated from the THP-1 monocyte cell line to each flame retardant for a period of 2 days in order to assess the compounds acute cytotoxic potential. While DOPO, EDA-DOPO, and PBDE-99 exhibited no signs of cell damage at concentrations as high as 400 µM, ETA-DOPO and EG-DOPO at concentrations >20 µM produced acute toxicity in both cell models (Fig. 2). These findings confirm observations reporting that PBDE-99 within the same concentration range used in the present study did not produce cell damage in A549 cells (Kim et al. 2014). Another report assessed DOPO toxicity in PC12 and B35 neuroblastoma cell models and also observed no damage using a concentration range comparable to that applied in the present study, further confirming the low cytotoxic potential of DOPO (Hendriks et al. 2014).

For cell types primarily involved in the inflammatory response, such as macrophages or astrocytes, previous reports indicated inflammatory activation in response to flame retardants (Koike et al. 2014). Therefore, we measured release of proinflammatory cytokines TNF-α and IL-8 in differentiated THP-1 macrophages and did not observe significant release (Suppl. Fig. 1a + b). It was recently reported that PBDE-47 and PBDE-209 induce oxidative stress and thereby contribute to DNA damage (Pellacani et al. 2012). In A549 cells, no significant increase in reactive oxygen species was observed (Suppl. Fig. 1c), nor were we able to detect DNA strand breaks in A549 or LUHMES cells exposed to the flame retardants tested in this work (Suppl. Fig. 2 + 3). As an alternative metric for the assessment of inflammatory activation evoked by the tested flame retardants, nuclear translocation of cytosolic subunits of the NF-κB complex was investigated in astrocytes and, similar to the results obtained in macrophages, no effect of the novel flame retardants was detected (Suppl. Fig. 4).

To allow direct comparison with published DOPO toxicity data in the same experimental model, the standardized acute daphnia immobilization (OECD guideline 202) test was employed. An EC_50_ of roughly 1.5 mM was recently reported for DOPO (Waaijers et al. 2013a, b). In our testing of EDA-DOPO, ETA-DOPO, and EG-DOPO, even the highest concentrations applied (water-soluble fraction of a 1000 mg/L/approximately 2 mM solution) resulted in minimal immobilization of 5–10 % of the treated daphnids (Suppl. Fig. 5). In an alternative OECD-approved assay (OECD guideline 201), freshwater microalgae were exposed to the flame retardants. At the highest tested concentrations (water-soluble fraction of a 1000 mg/L; equalling approximately a 2 mM solution), EG-DOPO inhibited algae growth, while EDA-DOPO and ETA-DOPO exhibited no effect (Suppl. Fig. 6).

Our observations confirm the low acute toxic potential of DOPO and its derivatives in non-neuronal cells and lower freshwater organisms, allow a comparison of our data on novel DOPO derivatives with other reports, and enable integration of the toxicological data presented herein into the network of data in the literature.

### Interaction of DOPO derivatives with epidermal barriers

**Fig. 3:**
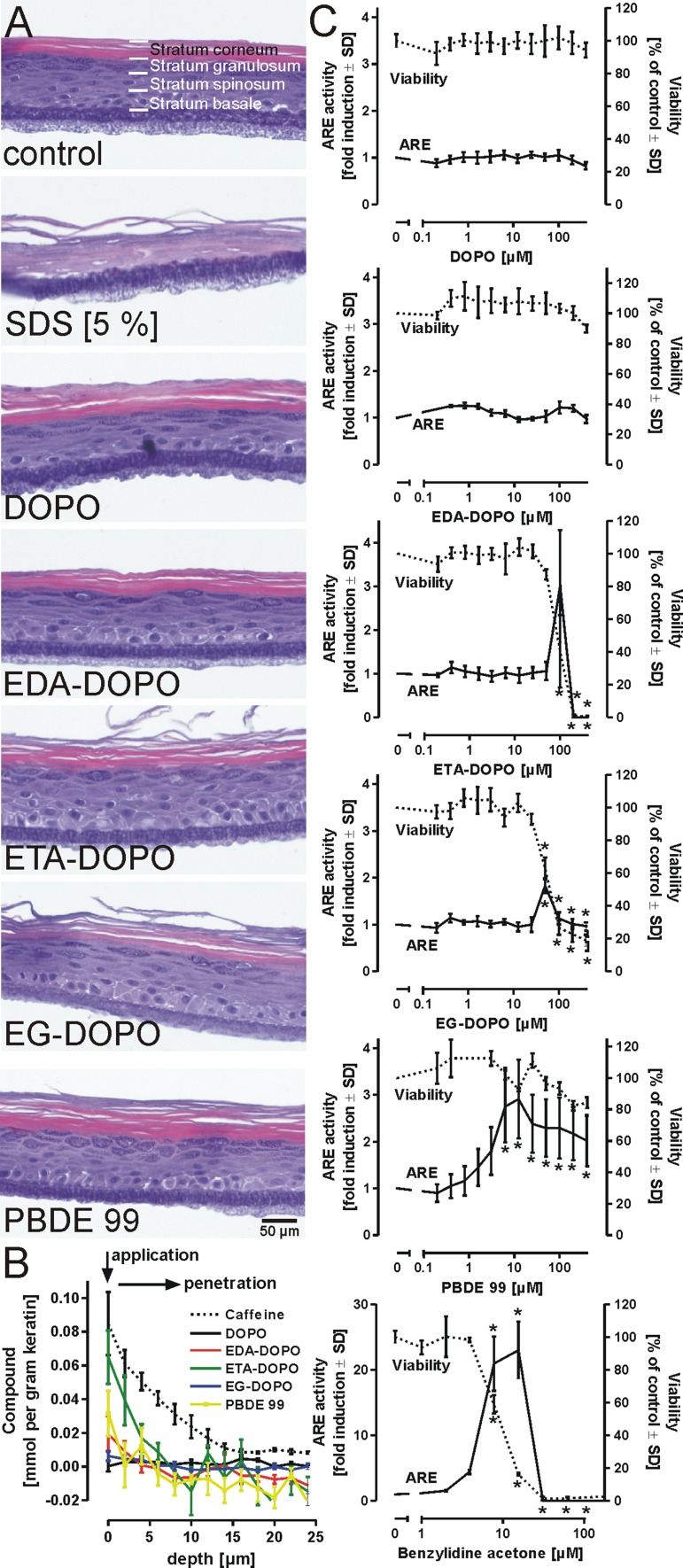
Interaction of DOPO derivatives with epidermal barriers. **a** Influence of flame retardants on epidermal integrity. The air-exposed stratum corneum of a 3D epidermal in vitro model generated from primary human keratinocytes was topically treated with various flame retardants (200 mM) for 15 min. The models were washed and further cultivated for 48 h. The tissues were analyzed by a viability assay and then fixed, sliced, and stained with hematoxylin and eosin to allow discrimination of epidermal layers. The loose layer beneath the stratum basale represents the filter membrane. **b** Penetration of flame retardants into skin. For the assessment of quantitative penetration profiles, porcine skin was topically exposed to the flame retardants (400 µM) for 1 h. Confocal Raman microscopy was applied to obtain a depth profile for each compound. The graph illustrates the amount of each flame retardant and that of positive control compound caffeine relative to keratin content as a function of depth. **c** Keratinocyte sensitization. To determine the skin sensitization potential of the tested flame retardants, KeratinoSens™ cells were stimulated with different concentrations of DOPO derivatives. Luciferase activity was detected 48 h after treatment, and the fold induction was calculated. Benzylidene acetone served as a positive control treatment. In parallel, the viability of the cells was assessed using the PrestoBlue^®^ reduction assay. All data are mean ± SD (*n* = 3); *p < 0.05

Dermal uptake of flame retardants present in ambient air and household dust, as well as by direct contact with plastic-containing materials, has emerged as a major uptake route in addition to ingestion and inhalation. To assess the effects of the novel flame retardants on the integrity of the skin, three different models were applied. For an analysis of the acute damage of the epidermal architecture, a 3D human in vitro epidermal model was utilized, in which samples were topically exposed to the respective flame retardant for 15 min at a concentration of 200 mM. Even at the highest concentrations applied to the stratum corneum, all tested flame retardants displayed no detectable influence on the architecture of the epidermis (Fig. 3a). Positive control compound 5 % SDS resulted in complete disintegration of the stratum corneum and substantial cell death in the stratum spinosum. The viability of the epidermal models was assessed using the PrestoBlue^®^ assay, which indicated no significant changes following exposure to the flame retardants (data not shown). The observed absence of significant epidermal damage could be the result of negligible penetration of the compounds into the epidermis or a consequence of epidermal cells having high resistance in comparison with that of other cell types. To assess penetration into a complex skin structure, the flame retardants were next applied topically to segments of porcine ears from freshly slaughtered animals. Uptake was detected in a noninvasive manner by application of confocal Raman spectroscopy (Fig. 3b). Caffeine was used as a positive control compound. The assays indicated significant accumulation of ETA-DOPO at the skin surface and moderate accumulation of PBDE-99 into porcine ear skin, while all other compounds displayed no detectable penetration into the skin within the investigated time period of 1 h.

### Assessment of the flame retardants sensitization potential

In order to assess the skin-sensitizer potential of the flame retardants when in direct contact with epidermal cells, a keratinocyte-based cell model optimized to detect electrophilic skin-sensitizing compounds was utilized. With this keratinocyte-based model, covalent modifications of active site cysteine residues in Keap1 by electrophiles lead to its dissociation from Nrf2, which subsequently accumulates in the nucleus and activates expression of genes with an antioxidant response element (ARE) in their promoter (Emter et al. 2010). Treatment of the reporter construct-carrying keratinocytes with the flame retardants revealed an increase in ARE-dependent luciferase expression following exposure to ETA-DOPO and EG-DOPO that was inversely correlated with a decline in cell viability. Under the experimental conditions employed in Fig. 3c, the increased strength of the luciferase signal cannot be attributed to a specific innate response of the cells, but must rather be interpreted as a consequence of cell death-associated processes. Comparable effects were observed with higher concentrations (>10 µM) of the positive control compound benzylidene acetone. However, PBDE-99 led to a concentration-dependent and moderate increase in ARE-dependent luciferase expression (ca. twofold increase at a concentration of 3 µM) in the absence of significant cell death, thus indicating the skin sensitization potential of PBDEs. DOPO and EDA-DOPO neither induced luciferase activity nor adversely affected cell viability (Fig. 3c), in agreement with the results shown in Fig. 2, which were obtained with human lung cells and macrophages. Taken together, these observations confirmed the cytotoxic potential of ETA-DOPO and EG-DOPO in 2D cell models.

Based on the assays applied herein, the tested flame retardants do not appear to have a direct adverse influence on epidermal integrity. There are also no reports indicating a direct degenerative influence of flame retardants present in household dust on human skin. More importantly, however, is the fact that some of the compounds have the potential to penetrate the stratum corneum and thus enter the human body. Our observations of DOPO derivative penetration were limited to only 1 h. Whereas uptake of ETA-DOPO and PBDE-99 was apparent even after this short incubation period, these experiments cannot exclude the possibility that DOPO, EDA-DOPO, and EG-DOPO might penetrate the skin and enter the human body under conditions of chronic interaction.

### Toxicity of flame retardants in central nervous system neurons

**Fig. 4:**
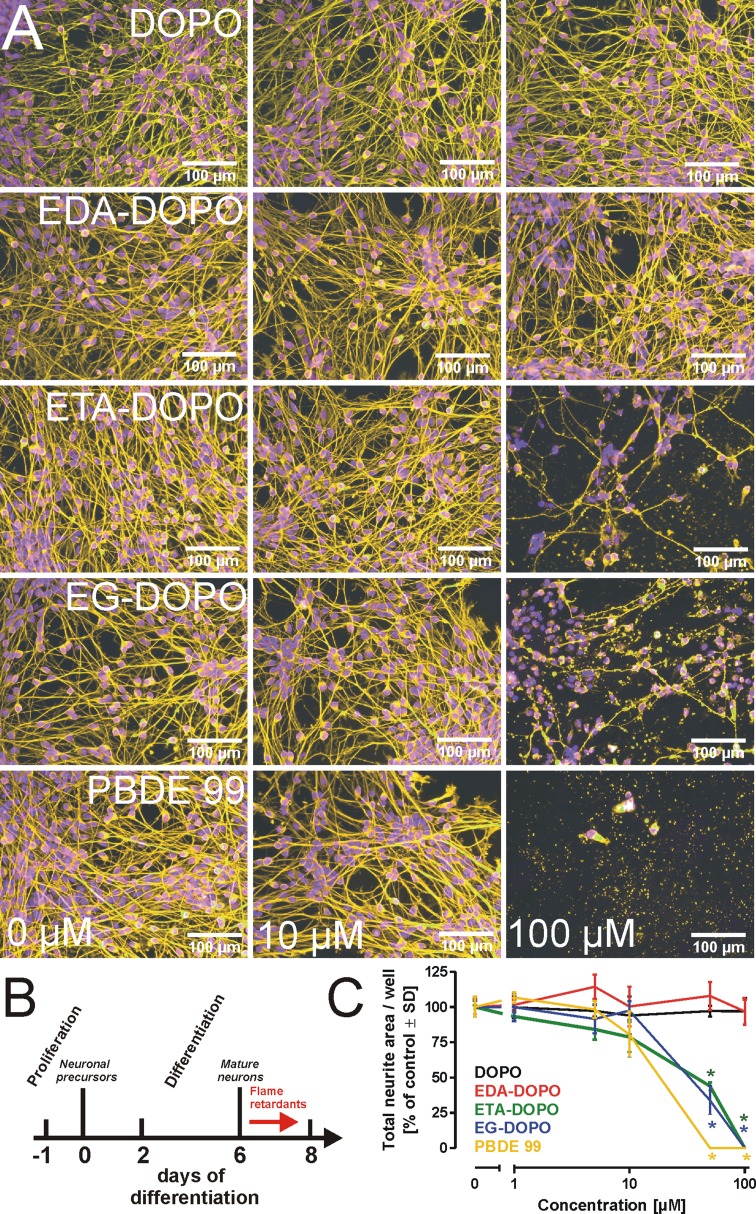
Toxicity in neurons of the central nervous system. **a** Mature human dopaminergic neurons (LUHMES) were treated with the flame retardants for 48 h from day 6 to day 8 of LUHMES differentiation as indicated in scheme (**b**). Cell morphology was visualized by fixation and staining of the cells with an anti-β-III-tubulin antibody (*yellow*), whereas nuclear DNA was stained with Hoechst dye H-33342 (*blue*). **c** For quantitative assessment of total neurite mass, cells were analyzed using an automated microscope system with an imaging algorithm that allowed cell bodies and cell extensions to be distinguished. The graph shows the total neurite mass detected following the respective treatments. All data are mean ± SD (*n* = 3); *p < 0.05

Previous studies on the toxicity of halogenated flame retardants identified a wide spectrum of different cell types and organs affected by exposure to such compounds, but they explicitly highlighted the adverse effects of halogenated flame retardants on neuronal integrity and neurodevelopmental processes (Hendriks et al. 2014). To investigate the influence of DOPO derivatives on fully differentiated central nervous system neurons, we exposed human LUHMES cells, which were differentiated into mature post-mitotic neurons within a period of 6 days (Schildknecht et al. 2009; Scholz et al. 2011). Fully differentiated LUHMES cells (day 6) were subsequently treated with varying concentrations of each flame retardant (Fig. 4a) for a period of 2 days (days 6–8; Fig. 4b). To visualize cell morphology, treated cells were fixed and stained with an anti-β-III-tubulin antibody. In our previous studies with LUHMES cells, we observed that neurite architecture served as a far more sensitive marker for neuronal integrity than did classical cell viability assays (Krug et al. 2013; Schildknecht et al. 2013). For the quantitative assessment of total neurite mass, an automated microscope system with an analysis algorithm allowing the detection of total neurite mass was applied (Fig. 4c). In addition, cell viability was detected by measuring the reduction of resazurin and by the lactate dehydrogenase (LDH) release assay, as well as by detecting intracellular levels of ATP and reduced glutathione (GSH) (Supp. Fig. 7). In summary, all assays indicated degeneration of neurites evoked by ETA-DOPO, EG-DOPO, and PBDE-99 at concentrations >10 µM, while DOPO and EDA-DOPO exhibited no influence at concentrations as high as 100 µM. These results were obtained with fully differentiated neurons. To investigate the influence of the flame retardants on neurite outgrowth when present during the differentiation process, LUHMES cells were treated from day 0 to day 2 of their differentiation period (Suppl. Fig. 8 and 9) that is characterized by the outgrowth of neurites. In the absence of acute cytotoxic effects, changes in neurite outgrowth are considered as a developmental neurotoxicity parameter. Qualitatively, the observations made in developing cells were the same as those made in fully differentiated cells, with the exception that EDA-DOPO also revealed a moderate, but significant influence on total neurite area. However, this effect was not reflected by intracellular levels of ATP and GSH, which served as cell viability markers (Suppl. Fig. 8 and 9). In order to introduce an alternative assay to investigate more subtle changes in neurite integrity, we investigated the migration of mitochondria in neurites of differentiated LUHMES cells (Suppl. Fig. 10). Treatment of the cells with the parkinsonian toxin MPP^+^, applied herein as a positive control compound, resulted in a significant decline in the velocity of mitochondrial migration in neurites, while all flame retardants exhibited no significant influence on mitochondrial migration under conditions where neurite integrity was not affected (25 µM, 12 h; Suppl. Fig. 10). At high concentrations, ETA-DOPO, EG-DOPO, and PBDE-99 influenced mitochondrial velocity; however, this effect was directly correlated with neurite degeneration and thus cannot be considered as an independent toxicity parameter.

Analysis of neurite mass and integrity in differentiating and mature LUHMES cells as neuro-selective readout and the comparison with results obtained by the resazurin reduction or LDH release assays as general cell viability markers, indicated that the tested flame retardants did not evoke neuro-specific degeneration. Instead, the observed impact on neurites could rather be considered as a consequence of general cell degenerative processes.

### Toxicity of flame retardants in neurons of the peripheral nervous system

**Fig. 5:**
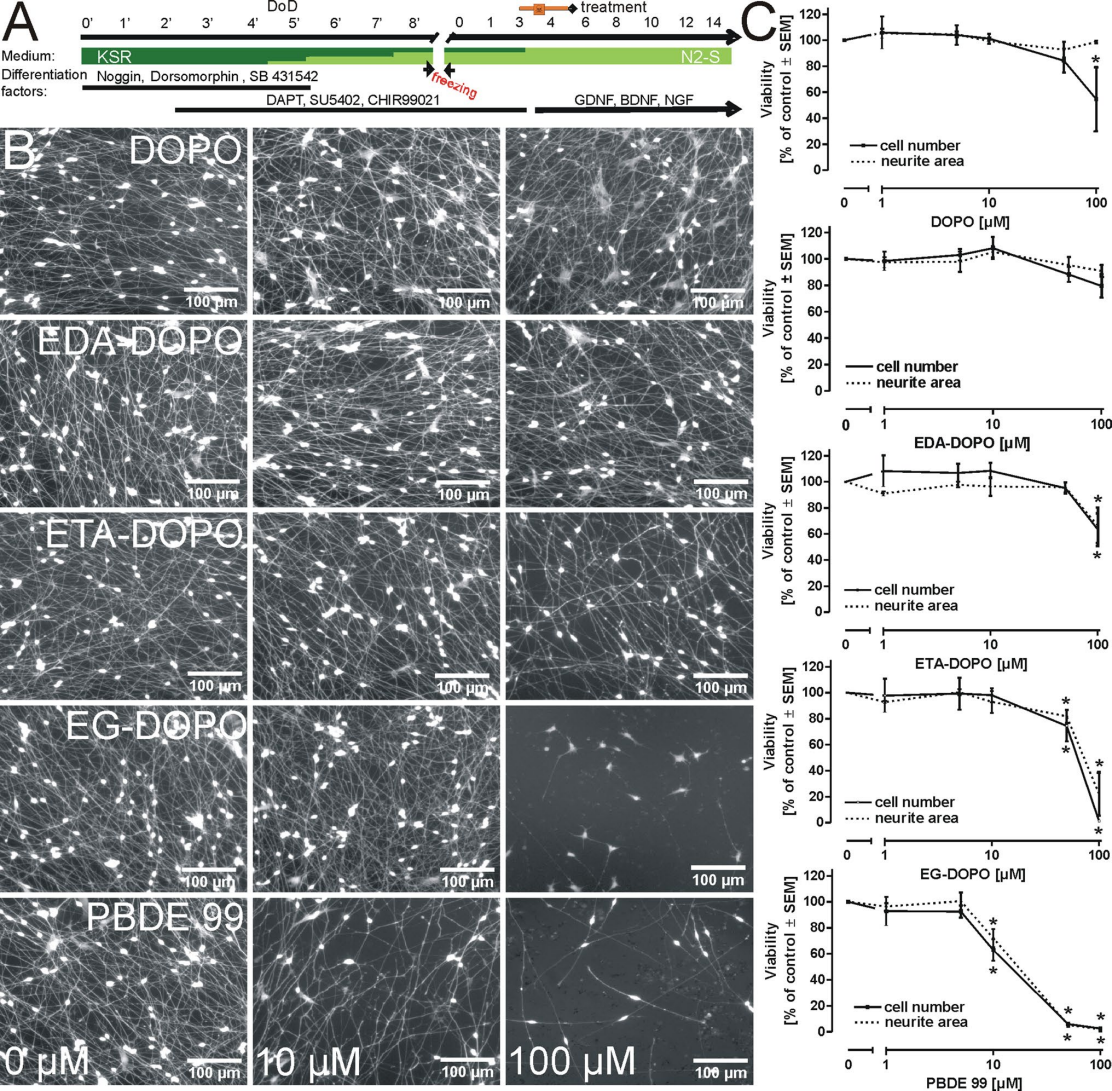
Toxicity in neurons of the peripheral nervous system. **a** Differentiation scheme for the generation of human dorsal root ganglia-like cells. The pluripotent stem cell line hESC-H9 was differentiated into peripheral neurons in a 2-step scheme. **b** Flame retardants were applied at day of differentiation (DoD) 3 post-thawing for a period of 48 h, after which neurite outgrowth was investigated by staining the cells with calcein-AM (cell morphology) and Hoechst dye H-33342 (nuclei). **c** Quantitative assessment of cell number and neurite mass was conducted with an automated microscope system. Image analysis was performed with optimized algorithms that allowed cells to be counted by counting nuclei. Neurite growth was quantitatively assessed by detection of the entire calcein signal subtracted from a defined area surrounding the nucleus, which was defined as the cell body. Data are mean ± SEM (*n* = 3); *p < 0.05

Neurons of the central nervous system (CNS) are protected by the blood-brain barrier. Therefore, it may be speculated that neurons of the peripheral nervous system represent a more vulnerable target because of their more direct exposure to environmental toxicants in vivo, in addition to intrinsic features distinguishing them from CNS neurons. To assess potential differences between central and peripheral neurons with respect to their responses to the tested flame retardants, we generated peripheral neurons from human stem cell-derived neural precursors and treated them with each flame retardant for 48 h (Fig. 5a). Similar to the observations made in CNS neurons, EG-DOPO and PBDE-99 significantly affected neurite integrity (Fig. 5b) and cell viability (Fig. 5c), while DOPO, EDA-DOPO, and ETA-DOPO moderately affected neurite mass and reduced cell viability at concentrations >50 µM. These observations indicate that no significant difference between central and peripheral neurons exists in vitro with respect to their damage in response to the tested flame retardants. Although we did not investigate the mechanisms underlying cell death, the parallel decline in neurite mass and cell viability observed in central and peripheral neurons, together with the comparable responses of non-neuronal cells (Figs. 2, 3), suggests that DOPO and its derivatives evoke a cell type-independent mechanism of cell damage.

### Influence of flame retardants on neural crest cell migration

**Fig. 6:**
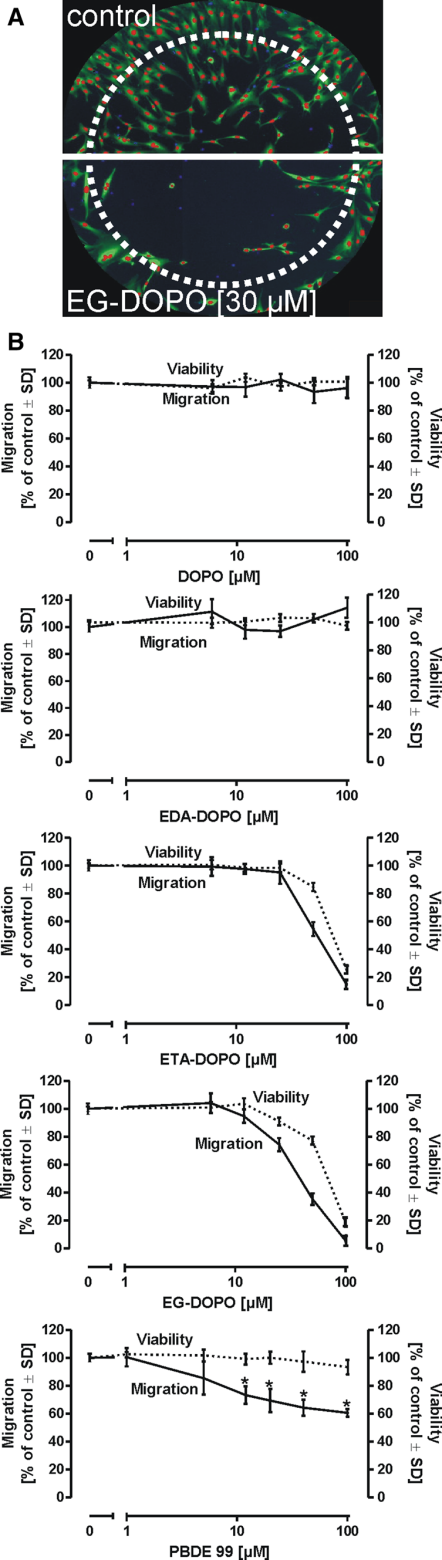
Influence of flame retardants on neural crest cell migration. **a** Neural crest cells were differentiated from the human embryonic stem cell line hESC-H9. The two images of control- and EG-DOPO-treated cells illustrate the general principle of the assay. The area inside the imaginary dotted ring was covered in the beginning of the experiment by silicone inserts, while the cells were seeded into the surrounding area. Following removal of the inserts, cells were allowed to migrate into the cell-free area. For the delineation and identification of single cells, nuclei were stained with Hoechst dye H-33342 (*blue*), whereas cell bodies were stained with calcein (*green*). For better visualization, areas of *blue* and *green* overlay are depicted in *red*. **b** Detection of migration and cell viability. Following removal of the stoppers, cells were allowed to migrate in the presence of the flame retardants for 24 h. The number of cells within the imaginary ring was counted and compared with the number of cells in the entire well to assess the contribution of cell proliferation events. Viability was detected by comparison of the number of calcein-positive cells with the total number of cells in the well. Data are mean ± SD (*n* = 3); *p < 0.05

The adverse effects of a compound on the nervous system are not necessarily correlated with its direct toxicity or its influence on neuronal differentiation. Even subtle changes in neural precursor migration during early development can profoundly influence the subsequent development of the nervous system. Therefore, we tested the flame retardants in a stem cell-derived human neural crest cell migration assay (Fig. 6). Cells were allowed to migrate into a cell-free area, as illustrated in Fig. 6a, after which migration and cell viability on the individual cell level were assessed. While DOPO and EDA-DOPO inhibited neither cell migration nor cell viability, ETA-DOPO and EG-DOPO both produced concentration-dependent declines in cell migration and cell viability (Fig. 6b). PBDE-99 moderately, but significantly, inhibited cell migration, but did not reduce cell viability. Similar effects were observed with ETA-DOPO and EG-DOPO in a relatively narrow concentration range of approximately 30–60 µM, in which migration was far more affected by these flame retardants in comparison with cell viability. This indicates an adverse effect of these compounds on early developmental processes. In all neuronal assays, ETA-DOPO and EG-DOPO showed cytotoxic properties at significantly lower concentrations than did DOPO and EDA-DOPO.

### Comparison and prioritization of organophosphorus flame retardants

**Fig. 7:**
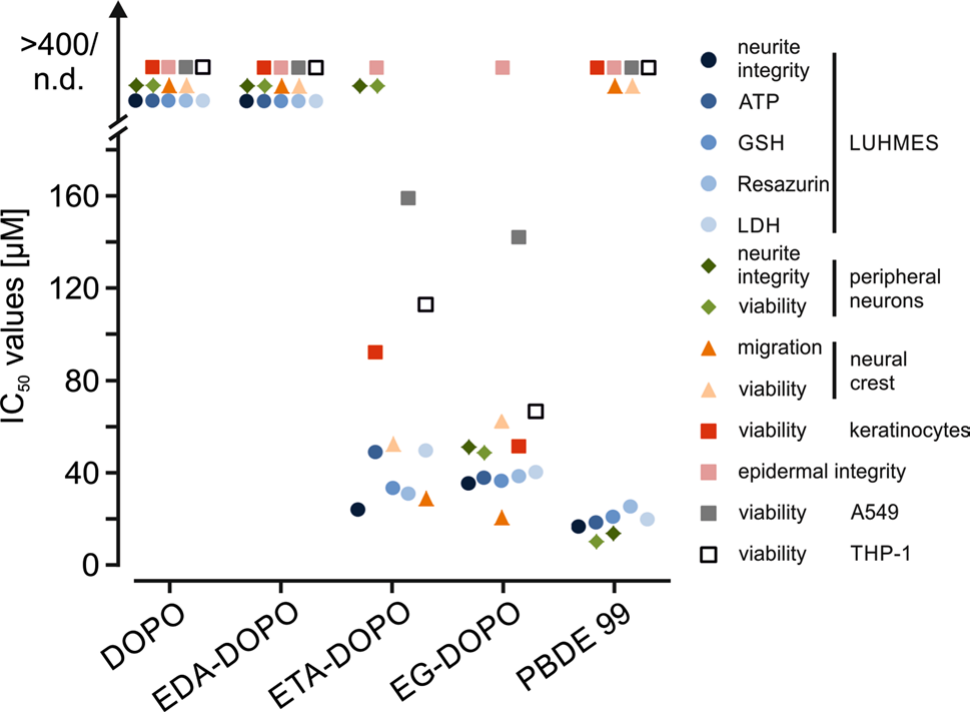
Synopsis of IC_50_ values. The plot summarizes the IC_50_ values obtained from the assays applied in this work. In the in vitro assays, maximal flame retardant concentrations were limited to 400 µM because of the maximal residual DMSO concentration of 0.1 %. Because of this limitation, IC_50_ values were not calculated for some compounds. The calculated IC_50_ values are illustrated on the lower segment of the plot. The data points at the *top of the plot* indicate that clear toxicity was not observed at test concentrations of 100–400 µM. *n.d.* not determined

In the present work, several in vitro test systems for the assessment of the potential adverse effects of the three novel DOPO derivatives were implemented. To allow a better comparison between the safety profiles of the tested compounds, the IC_50_ values determined by these assays were plotted in a single graph (Fig. 7). Because of the limited solubility of the compounds at maximal residual DMSO levels of 0.1 %, the maximal concentration in two-dimensional cell culture experiments was limited to 400 µM. Within this concentration range, some assays displayed no reduction in viability, precluding calculation of IC_50_ values. In all assays utilized, EDA-DOPO exhibited no harmful effects, while the average IC_50_ values of ETA-DOPO and EG-DOPO were in the range of 20–100 µM in all applied models. The three novel DOPO derivatives tested in the present work all exhibited superior flame retardation potential in comparison with that of their parental compound DOPO. The present initial analysis showed the cell damaging potential of ETA-DOPO and EG-DOPO within a concentration range comparable to that at which PBDE-99 produces toxicity. EDA-DOPO displayed flame retardation superior to that of its parental compound DOPO and evoked no harmful effects in any assay employed in this work.

## Discussion

### Acute toxicity of DOPO derivatives in context with current literature data

In the present work, the potential harmful influence of novel halogen-free *bis*-DOPO compounds linked via alkyl spacers by N- and O-bonds was assessed for the first time using a set of cellular in vitro assays. The three novel compounds were designed in order to possess improved flame retardation potential, but also for a wide range of applications based on their higher melting points and increased stability, which are prerequisites for their use in the production of plastic materials (Buczko et al. 2014; Gaan et al. 2013, 2015; Neisius et al. 2014; Salmeia and Gaan 2015; Salmeia et al. 2015; Qiang et al. 2011). In contrast to the current practice of investigating the potential harmful properties of flame retardants after their introduction into the market, we applied here a series of human in vitro screening assays to allow a fundamental toxicological classification before the production and application of halogen-free *bis*-DOPO compounds on an industrial scale. In contrast with the vast literature on polybrominated diethyl ethers, covering aspects such as indoor and outdoor contamination levels, human uptake routes, accumulation in different organs and tissues, and toxic effects in a variety of in vitro and in vivo test systems (Watkins et al. 2011; Stapleton et al. 2014; Lyche et al. 2015; Liagkouridis et al. 2015), only limited data on DOPO toxicity, and no information on the harmful influence of *bis*-DOPO derivatives, are available.

In order to allow a direct comparison of the toxicological data presented in this study with published data on polybrominated diphenyls, the parental compound DOPO and PBDE-99, as a representative PBDE, were incorporated in all experiments. At concentrations up to 400 µM, DOPO displayed no influence on all parameters investigated herein. These findings verify data from a previous study by Hendriks et al., in which rat adrenomedullary pheochromocytoma PC12 cells and rat neuroblastoma B35 cells that were treated for 24 h with the maximal concentration of 100 µM DOPO exhibited no signs of alterations in cell viability, generation of reactive oxygen species, or intracellular Ca^2+^ levels (Hendriks et al. 2014). In a standardized daphnia immobilization assay (OECD Guidelines for the Testing of Chemicals, Test No. 202), treatment of young daphnids with the water-soluble fraction of a 1000 mg/L suspension (approximately 2 mM) of DOPO for 48 h did not significantly influence viability (Waaijers et al. 2013a, b). Our toxicological assessment of the three *bis*-DOPO compounds with the same standardized daphnia model revealed no significant influence at any concentration applied (water-soluble fraction of a 1000 mg/L suspension; Suppl. Fig. 5). Based on this information, we applied DOPO as a negative control reference compound in our experiments to allow direct comparison with currently available data and to enable integration of the information generated in the present work into the existing network of toxicological data on flame retardants. PBDE-99, a representative polybrominated diphenyl compound, was chosen as a positive control. Among the vast number of PBDE variants produced, five congeners, PBDE-47, PBDE-99, PBDE-100, PBDE-153, and PBDE-154, predominate in human tissues and account for more than 90 % of PBDEs detected in the human body (Stasinska et al. 2014). The reported absence of adverse effects by PBDE-99 in non-neuronal cells at concentrations up to 100 µM was confirmed by our observations made with A549 cells, THP-1 cells, and keratinocytes (Figs. 2, 3). However, epidemiological investigations focusing on the relationship between PBDE exposure in early development and deficits in psychomotor and IQ performance in later life indicated a profound influence of PBDE-99 on the development and function of the neuronal system (Shy et al. 2011; Gump et al. 2014; Herbstman et al. 2010; Chevrier et al. 2010; Chao et al. 2011). Our observation of the selective influence of PBDE-99 on central and peripheral neuronal integrity was fundamentally supported by other reported in vitro observations indicating pronounced selective toxicity in neuronal cells (Schreiber et al. 2010). These observations clearly indicate that the cell type-selective toxicity of PBDEs is adequately represented by the in vitro models employed in the present study. Combined with the observations made with DOPO, which served as the negative control compound in our work, the information on the toxicological response to PBDE-99 allows direct comparison with the relatively large number of in vitro observations in the literature. Data on PBDEs in the literature allow some correlation between their toxicity in cell culture in vitro systems, their levels in different human organs and tissues, and their adverse effects on psychomotor functions in humans. Under the assumption of comparable volatilization rates and bioaccumulation properties of PBDEs and *bis*-DOPO compounds, the data presented herein allow preliminary speculation on the potential interference of *bis*-DOPO compounds with neurodevelopmental processes in vivo, which should be addressed by utilizing in vivo test models to compile an adverse effect profile of these compounds.

### Comparison of in vitro and in vivo flame retardant burdens

Toxicological results obtained with in vitro models raise the question of actual in vivo concentrations in humans, which are largely influenced by the biophysical properties of a given compound. Comprehensive literature on indoor and outdoor contamination levels and organ and tissue accumulation rates is currently available for PBDEs. Several studies analyzing PBDE levels in cohorts in various countries have been published in recent years (Chao et al. 2007; Roze et al. 2009; Eskenazi et al. 2013). Exposure of infants to PBDEs has been routinely observed as significantly higher than that of adults, largely as a result of higher rates of oral, nasal, and dermal uptake, as well as of different toxicokinetic parameters (Toms et al. 2009; Rose et al. 2010). PBDE-99 was identified in office dust at concentrations of approximately 900 ng/g dust (Watkins et al. 2013). While uptake of house dust by adults was found to be in the range of 20–50 mg/day, that of smaller children was found to reach 100–200 mg/day (Wilford et al. 2005; U.S. EPA 2008). For children, this rate of dust uptake implies a daily uptake of PBDE-99 in the range of 75 ng/day. Based on the lipophilicity of PBDEs and their ability to pass the placental barrier, breastfed infants are exposed to a daily oral dose of PBDE congeners approximately 300-fold higher than that to which adults are exposed (Toms et al. 2009; Rose et al. 2010; Costa et al. 2014). Average blood levels of PBDEs in adults in the USA have been reported in the range of 10–300 ng/g lipid, whereas the reported infant blood levels of PBDEs were almost an order of magnitude greater (Costa and Giordano 2007; Toms et al. 2009; Rose et al. 2010). Assuming a uniform distribution of PBDEs in the blood, these numbers equate to a steady-state PBDE concentration in the range of 10–100 nM in the blood (Costa et al. 2014). Considering their lipophilicity, accumulation of PBDEs in lipid-rich tissues such as the brain, kidneys, liver, and fat can easily lead to local concentrations in the low micromolar range, which is similar to the range of concentrations tested in this work.

### Epidermal uptake of organophosphorus flame retardants and evaluation of sensitization effects

The primary routes of flame retardant uptake have been generally considered to be ingestion and inhalation. However, recent pharmacokinetic calculations indicate that dermal uptake represents the second most important contributor to the PBDE body burden, exceeded only by uptake via ingestion (Abdallah et al. 2015). DOPO and its *bis*-DOPO derivatives are additives and thus are not covalently bound to their host material, implying an inherent tendency for volatilization. Considering their primary use in plastic materials used in everyday consumer products such as household appliances and furniture, dermal contact with DOPO derivatives is an important health concern following their introduction onto the market. We therefore investigated the influence of DOPO derivatives on the integrity of a three-dimensional in vitro epidermal model, as well as their sensitization potential and cytotoxicity when in contact with cultured keratinocytes. Apical application of flame retardants at concentrations of up to 200 mM (in DMSO) resulted in no apparent disintegration of the epidermal architecture. The stratum corneum represents the outermost layer of the epidermis and consists of nucleus- and organelle-free corneocytes that are constantly replaced by desquamation. Direct contact of the flame retardants with the stratum corneum apparently had no influence on its integrity (Fig. 3a), but evoked the question of the influence of the flame retardants when in contact with organelle-containing cells of the stratum granulosum or stratum spinosum. In order to address this question, we first analyzed the depth to which apically applied flame retardants can penetrate an intact epidermis. Analysis by Raman microscopy indicated detectable accumulation of ETA-DOPO and to a lesser extent of PBDE-99 at the porcine skin surface with a maximal penetration depth of less than 8 µm. Human stratum corneum has a typical thickness of 17 µm, while the thickness in pigs is 21–26 µm (Godin and Touitou 2007). These findings indicate that penetration of externally applied flame retardants can be efficiently deferred by the stratum corneum; however, the incubation interval employed in Fig. 3b was limited to only 1 h, which contrasts with the chronic exposure of humans to flame retardants under everyday conditions. The assessment of the toxicity of EDA-DOPO, ETA-DOPO, and EG-DOPO in a two-dimensional keratinocyte cell culture model revealed IC_50_ values (Fig. 3c) for the flame retardants that were comparable to our observations in macrophages and lung epithelial cells (Fig. 2). In order to evaluate whether the flame retardants may act as sensitizers on human skin, the KeratinoSens™ assay that primarily assesses the reaction of compounds with cysteine residues was performed. No specific effects that would classify DOPO, EDA-DOPO, ETA-DOPO, or EG-DOPO as sensitizers were apparent. These high concentrations applied in this work are rather unlikely to occur by exposure to ambient air and household dust. Furthermore, in a living organism, penetrated compounds are continuously distributed by the vascular system. By taking these considerations into account, it can be concluded that the direct harmful influence of the flame retardants tested in this study on epithelial barriers is likely to be negligible. However, the observed import of extracorporeal flame retardants via the skin apparently represents a potential route for their uptake, which is an essential parameter in the evaluation of their safety profile.

### Neurotoxic potential of DOPO derivatives

Studies on the harmful potential of polybrominated flame retardants highlighted their explicit adverse influence on the neuronal system (Costa and Giordano 2007; Costa et al. 2014). Exposure of test animals to PBDEs during early development slowed motor skill development, influenced synaptic plasticity, and altered hippocampal long-term potentiation in rats and mice (Viberg et al. 2006; Dingemans et al. 2007; Ta et al. 2011). Recent human epidemiological studies confirmed the relationship between exposure to PBDEs during early development and reduced psychomotor development, motor function, and IQ in childhood (Chevrier et al. 2010; Herbstman et al. 2010; Chao et al. 2011; Gump et al. 2014). Plasma levels of PBDEs detected in these studies were usually not associated with acute neurodegenerative events. However, early brain development is characterized by phases of precursor proliferation, migration, and differentiation that are tightly regulated spatially and temporally. Therefore, it can be assumed that moderate interference by environmental toxicants during early brain development might lead to miswiring of neuronal circuits or changes in the ratios of different neuronal types. Such an effect would lead to increased levels of particular neurotransmitters and reduced levels of others, as shown by inhibitory effects on the cholinergic system, which is involved in learning and memory (Viberg et al. 2003).

Based on the literature, we applied a series of in vitro test systems to study the potential harmful influence of organophosphate flame retardants on the integrity of differentiated and differentiating neurons of the central and peripheral nervous system, as well as their influence on NCC migration. In our previous studies on in vitro neurodegeneration, we utilized quantitative assessment of total neurite mass and migration of mitochondria in neurites as metrics with greatly improved sensitivity in comparison with that of standard cytotoxicity assays such as resazurin reduction, MTT reduction, or the LDH release assay. Interestingly, neurite integrity and mitochondrial migration occurred in a concentration- and time-dependent manner that closely reflected the decline in central and peripheral neuron viability detected by resazurin reduction, LDH release, and uptake of calcein-AM (Figs. 4, 5; Suppl. Fig. 10). Furthermore, the concentration-dependent toxicity evoked by ETA-DOPO and EG-DOPO in the neuronal models occurred at a lower concentration range than that which produced toxicity in the non-neuronal models (Figs. 2, 3). In both models of central and peripheral nervous system neurons, comparable profiles of cell damage and toxicity were observed in fully differentiated cells and differentiating cells (Fig. 4; Suppl. Fig. 7, 8, and 9). In contrast, and in agreement with previous reports, PBDE-99 exhibited distinct neurotoxic effects and detrimentally influenced central and peripheral neurons at concentrations >10 µM, while the compound produced no significant toxicity in non-neuronal models such as macrophages, lung epithelial cells, and keratinocytes at concentrations as high as 400 µM. These findings clearly illustrate that DOPO and its derivatives possess a cell type-independent toxicity profile. Previous reports on the damage evoked by PBDEs in the nervous system highlight their influence on neuronal differentiation and neural precursor cell migration (Zimmer et al. 2014). Inappropriate migration of precursor cells in early brain development would entail significant disturbance in neuronal function. DOPO and EDA-DOPO influenced neither NCC migration nor NCC viability at concentrations as high as 100 µM. ETA-DOPO and EG-DOPO affected cell viability in a concentration range identical to that used to obtain the results shown in Figs. 2, 3, 4, and 5. Interestingly, in contrast with the effects of PBDE-99 on differentiating and fully differentiated central and peripheral neurons (Figs. 4, 5), PBDE-99 did not reduce the viability of human NCCs at concentrations as high as 100 µM (Fig. 6). However, migration of NCCs was inhibited by PBDE-99. These observations fully corroborate the findings of a previous report utilizing NCCs (Zimmer et al. 2014), in which PBDE-99 inhibited migration in the absence of cell death at concentrations as high as 100 µM. An investigation of the selective neurotoxicity of PBDE-99 was outside the scope of the present work; however, the data provided herein and in the literature indicate an interaction of PBDE-99 with neuro-selective targets that are expressed after the migration of immature neurons to their ultimate position in the brain, but before neurite outgrowth is inhibited.

## Conclusion

The present work represents the first toxicological assessment of DOPO derivatives EDA-DOPO, ETA-DOPO, and EG-DOPO with an explicit focus on cellular in vitro models. The comparison with PBDE-99 as a representative polybrominated diphenyl ether convincingly demonstrated that ETA-DOPO and EG-DOPO possess cell type-independent cytotoxic properties at concentrations >10 µM, while DOPO and EDA-DOPO display no comparable effects at concentrations higher than 100 µM, as can be deduced from the graphical summary of all IC_50_ values calculated from the experiments reported herein (Fig. 7). The information provided herein allows targeted design of in vivo experiments addressing questions regarding uptake, distribution, and accumulation of DOPO derivatives in organs and tissues, as well as their influence on developmental processes. Combination of the present in vitro data with in vivo observations in test animals would provide a solid platform for a qualified initial assessment of the adverse potential of DOPO derivatives EDA-DOPO, ETA-DOPO, and EG-DOPO in humans.

## Electronic supplementary material

Below is the link to the electronic supplementary material.

Supplementary material 1 (PDF 2588 kb)
