# Supplementary material for: Multiparameter toxicity assessment of novel DOPO-derived organophosphorus flame retardants
Source: Arch Toxicol. 2016 Feb 29;91(1):407–25. doi: 10.1007/s00204-016-1680-4 (PMC5225203; doi:10.1007/s00204-016-1680-4)
Supplement: Supplementary file 1 — Supplementary material 1 (PDF 2588 kb) [file 204_2016_1680_MOESM1_ESM.pdf]

Supplementary Material

Multiparameter toxicity assessment of novel DOPO-derived organophosphorus flame retardants

Cordula Hirsch[1*], Britta Striegl[3], Stephanie Mathes[3], Christian Adlhart[3], Michael Edelmann[3], Epifania Bono[3], Sabyasachi Gaan[4], Khalifah A. Salmeia[4], Lisa Hoelting[2], Alice Krebs[2], Johanna Nyffeler[2], Regina Pape[2], Alexander Bürkle[2], Marcel Leist[2], Peter Wick[1], Stefan Schildknecht[2]

[1]Particles-Biology Interactions Laboratory, Swiss Federal Laboratories for Materials Science and Technology (Empa), Lerchenfeldstrasse 5, CH-9014 St. Gallen, Switzerland
[2]University of Konstanz, Universitaetsstr. 10; 78457 Konstanz, Germany
[3]ZHAW; Life Sciences and Facility Management; Einsiedlerstr. 31; 8820 Waedenswil, Switzerland
[4]Additives and Chemistry Group, Advanced Fibers, Swiss Federal Laboratories for Materials Science and Technology (Empa), Lerchenfeldstrasse 5 CH-9014, St. Gallen, Switzerland

*To whom correspondence should be addressed:

Cordula Hirsch, PhD
Particles-Biology Interactions Laboratory
Swiss Federal Laboratories for Material Science and Technology (Empa)
Lerchenfeldstrasse 5
CH-9014 St. Gallen, Switzerland
Tel. +41 58 765 7791
Fax. +41 58 765 7499
Email: Cordula.Hirsch@empa.ch

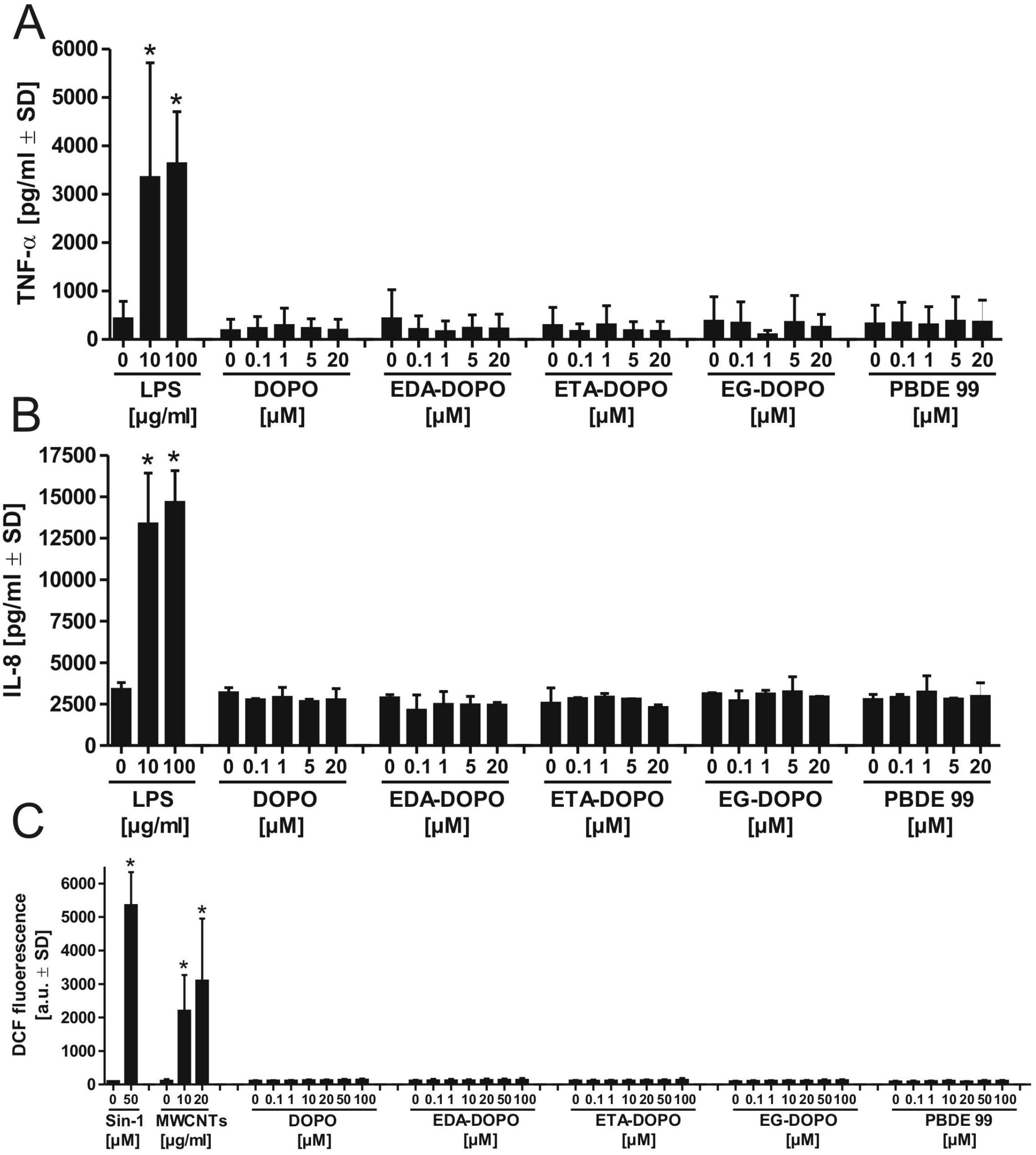

Suppl. Fig 1. Inflammatory activation potential of organophosphorus flame retardants. Human monocytic THP-1 cells were seeded at a density of 4 x 10^4 cells/well in a 96-well plate and differentiated into mature macrophages by phorbol 12-myristate 13-acetate (PMA) (200 nM) for 3 days. The cells were then treated with the respective flame retardants in the concentrations as indicated for 8 h, lipopolysaccharide (LPS) was added as positive control. The amount of TNF-α (a) and IL-8 (b) in the supernatant was detected by ELISA (eBioscience, Vienna, Austria). (c) For the detection of reactive oxygen species formed in response to flame retardant treatment, human lung epithelial A549 cells were seeded at a density of 2 x 10^4 cells/well in 96-well plates and grown for 1 day. Then, cells were loaded with H_2DCF-DA (50 µM) for 1 h in Hanks Balanced Salt Solution (HBSS). After two washing steps, the cells were treated for 2 h with the respective flame retardants. The peroxynitrite-generating compound Sin-1 (50 µM) and multi-walled carbon nanotubes (MWCNTs, 10 and 20 µg/ml) served as positive controls. Data are mean ± SD (n = 5) *$p < 0.05$.

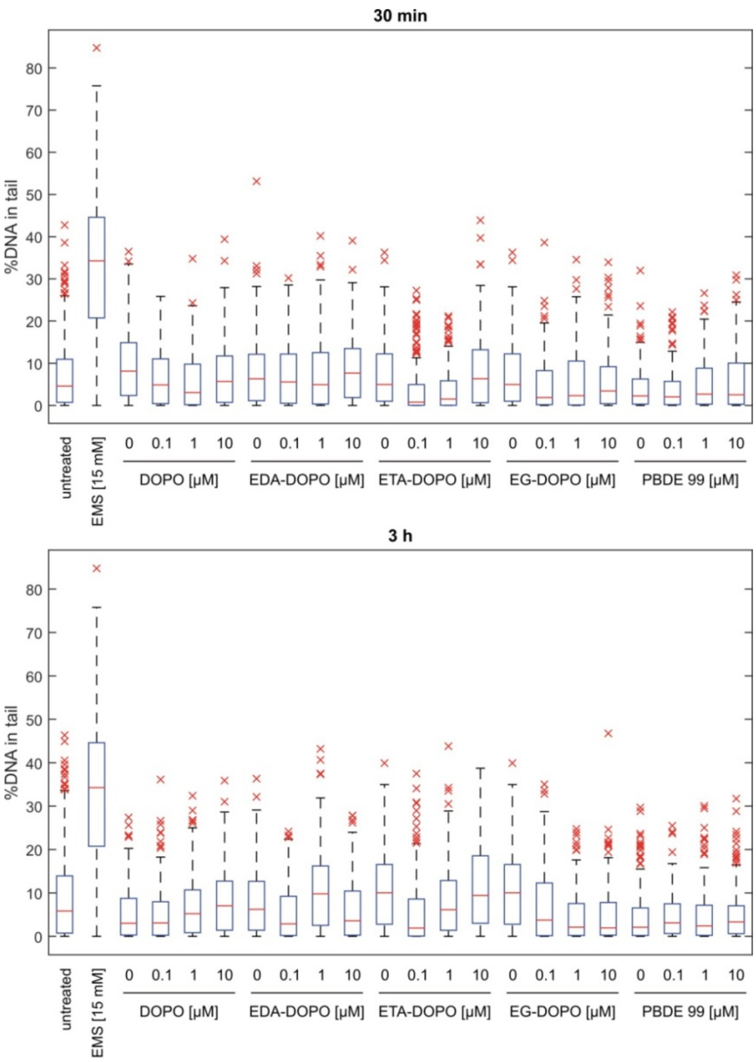

Suppl. Fig. 2. Detection of DNA strand breaks using the alkaline Comet assay. Human lung epithelial cells (A549) were seeded into 6-well plates at a density of 2.5 x 10^5 cells/well and grown for 1 day. The cells were then treated with the respective flame retardants for 30 min or 3 h. Ethylmethanesulfonate (EMS) (15 mM) served as positive control. The assay was performed according to Singh et al. Exp. Cell Res. 1988; 175: 184-191. DNA was stained with ethidiumbromide (20 µg/ml) and comets were analyzed using a Nikon Eclipse TS 100 microscope equipped with a Stingray F046B IRF Jenofilt camera and "Comet Assay IV" software (Perceptive Instruments, Haverhill, UK). Samples were blinded and 100 randomly chosen comets per slide were analyzed.

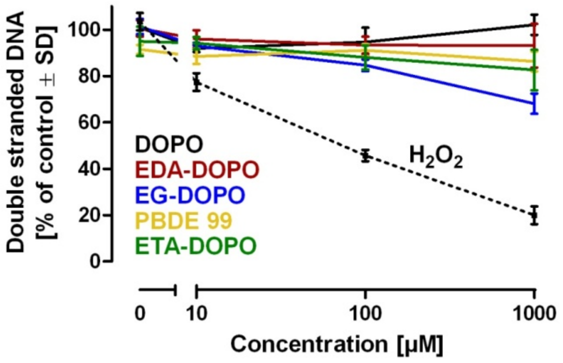

Suppl. Fig. 3. Detection of DNA strand breaks by the Fluorimetric detection of Alkaline DNA Unwinding (FADU) assay. LUHMES were trypsinized and resuspended in Advanced DMEM/F12 medium at a density of 2 x 10^6 cells/ml. Cells in suspension were then incubated with the respective flame retardants for 3 h at 37°C. DNA strand breaks were analysed by an automated FADU assay based on progressive DNA unwinding under defined conditions of alkaline pH and analysis of DNA unwinding via detection of remaining double-stranded DNA by Sybr®Green as previously described in detail in Moreno-Villanueva et al. ALTEX 2011; 28: 295-303.

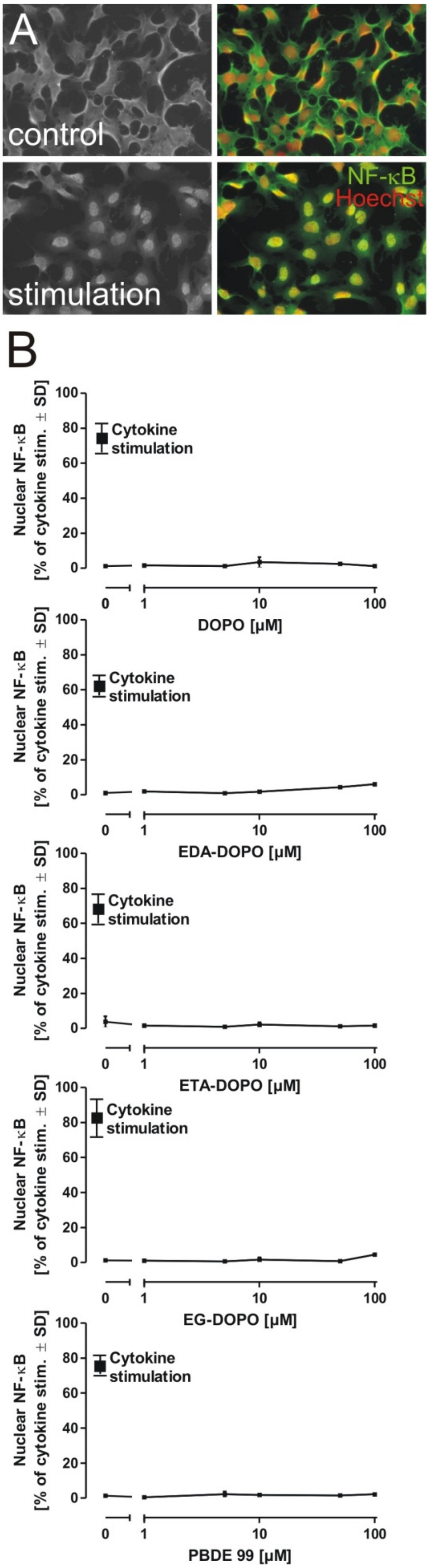

Suppl. Fig. 4. Nuclear translocation of NF-κB. IMA 2.1 astrocytes were seeded into 96 well plates at a density of 10.000 cells/well and were allowed to grow for one day. The cells were then treated with the respective flame retardants as indicated. As positive control, cells were stimulated with a cytokine mix consisting of TNF-α (10 ng/ml), IL-1β (10 ng/ml), and IFN-γ (20 ng/ml) for 30 min. Cells were then fixed and stained with an anti-NF-κB p65 specific antibody. Images of the translocation of NF-κB from the cytosol to the nucleus (stained by Hoechst H-33342) were quantitatively assessed by forming the ratio between the mean average staining intensity in the nuclear area and the mean average signal intensity of a defined "ring" surrounding the nucleus. Values are depicted as percentage of total NF-κB staining intensity.

EDA-DOPO

Loading rate (mg/l)	Replicate	Number of individuals per test vessel	Immobile *Daphnia* after 48 h [in %]
0	A<br>B	20<br>20	0<br>0
10	A<br>B	20<br>20	0<br>0
100	A<br>B	20<br>20	0<br>0
1000	A<br>B	20<br>20	5<br>10

ETA-DOPO

Loading rate (mg/l)	Replicate	Number of individuals per test vessel	Immobile *Daphnia* after 48 h [in %]
0	A<br>B	20<br>20	0<br>0
10	A<br>B	20<br>20	5<br>0
100	A<br>B	20<br>20	0<br>0
1000	A<br>B	20<br>20	5<br>0

EG-DOPO

Loading rate (mg/l)	Replicate	Number of individuals per test vessel	Immobile *Daphnia* after 48 h [in %]
0	A<br>B	20<br>20	0<br>0
10	A<br>B	20<br>20	0<br>0
100	A<br>B	20<br>20	10<br>0
1000	A<br>B	20<br>20	5<br>10

Suppl. Fig. 5. Freshwater microcrustacean toxicity test. Acute toxicity was determined in Daphnia magna according to the standardized OECD guidelines number 202 test, performed by BMG Engineering Ltd., Schlieren, Switzerland. Daphnia were cultured in aerated Elendt M4 medium at a constant temperature of 21°C under a 16:8 light/dark photoperiod. The water soluble fraction of the indicated concentrations of flame retardants were added for 48 h. Mortality was assessed by the detection of movement analysis. Immobile Daphnia were defined as those animals displaying no movement within a 15 s period following shaking of their glass flasks.

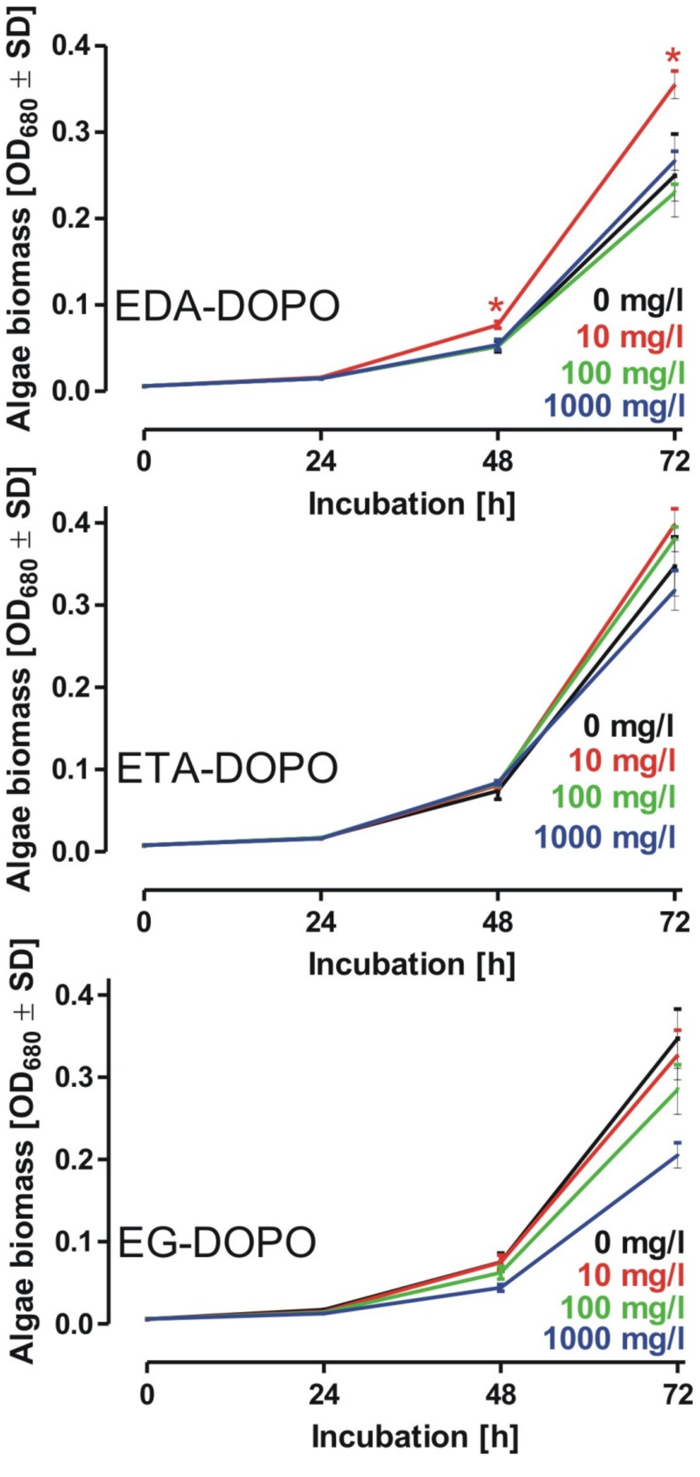

Suppl. Fig. 6. Freshwater microalgae toxicity test. Acute toxicity was tested by assessing algal growth inhibition applying Desmodesmus subspicatus according to OECD guideline number 201. Growth inhibition tests were performed using an initial cell density of 5 x 10^3 cells/ml (= OD_{680} of 0.005). The cells were maintained in 250 mL flasks containing 100 mL of sterile OECD algal medium at 22°C at 130 rpm illuminated with 3000 lux. Cell density was detected photospectrometrically at λ_{680} nm in 24 h intervals in algae cultures treated with the respective flame retardants. Experiments were performed by BMG Engineering Ltd., Schlieren, Switzerland.

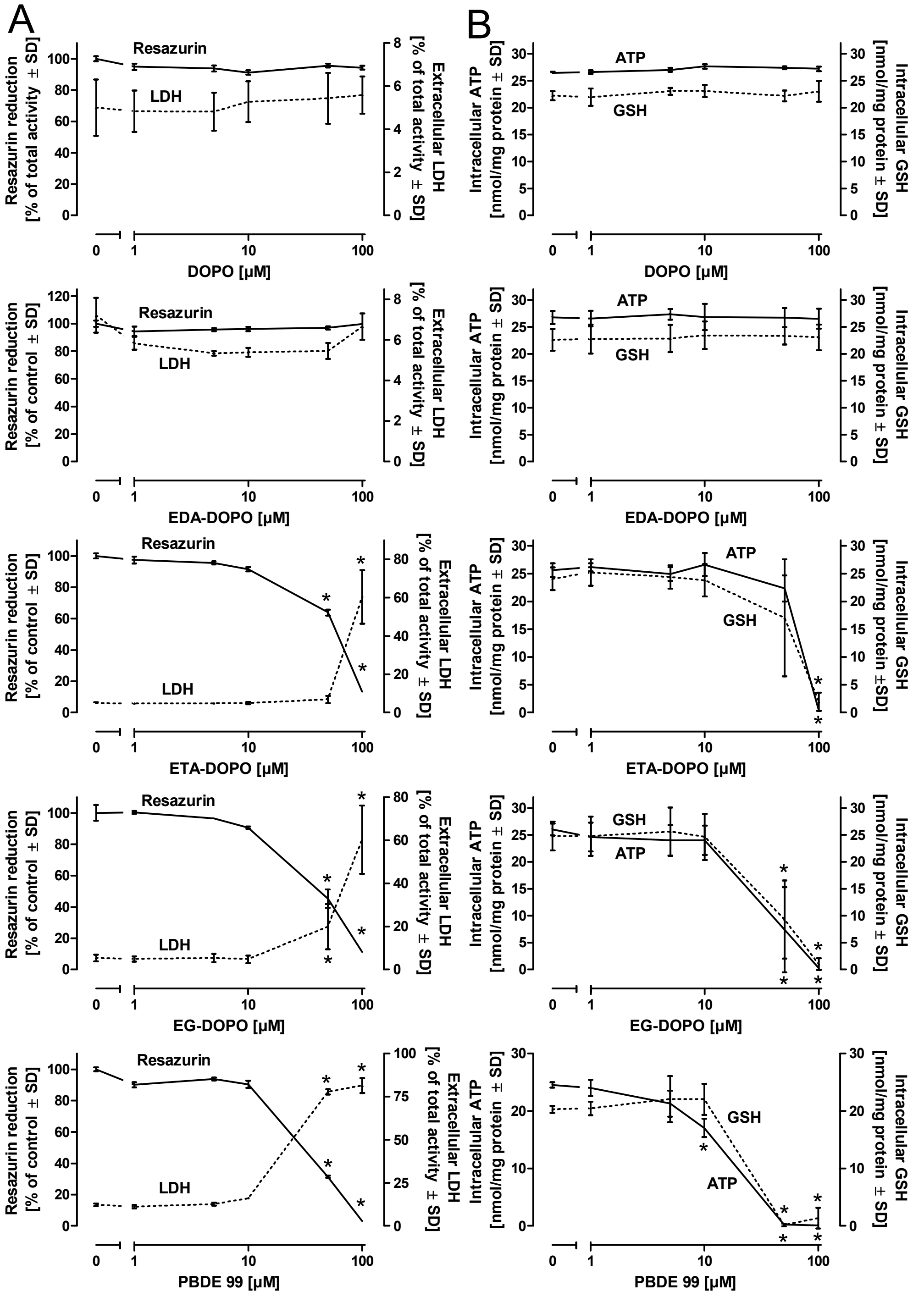

Suppl. Fig. 7. Toxicity of flame retardants in central human neurons. Mature human dopaminergic neurons (LUHMES) were treated with the different flame retardants in the concentrations as indicated for 48 h from day 6 to day 8 of differentiation. **a** Cell viability was determined by the resazurin reduction and by the lactate dehydrogenase release assays. **b** Intracellular levels of ATP and of reduced glutathione (GSH) were measured in parallel.

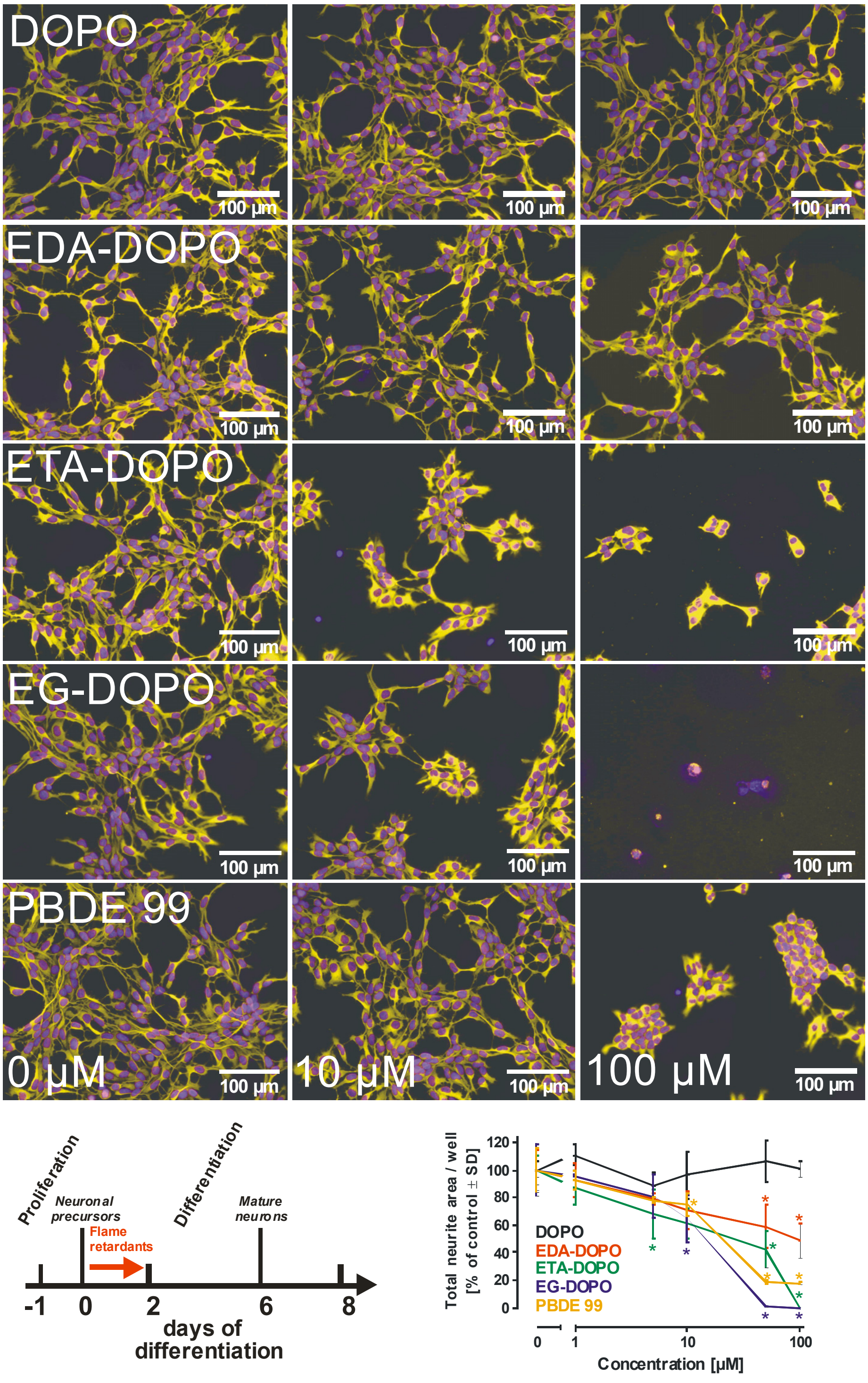

Suppl. Fig. 8. Influence of flame retardants on the integrity of central human neurons. Differentiating human dopaminergic neurons (LUHMES) were treated with the different flame retardants in the concentrations as indicated for 48 h from day 0 to day 2 of LUHMES differentiation. Cell morphology was visualized by fixation and staining of the cells with an anti-β-III-tubulin antibody (yellow), nuclear DNA was stained with Hoechst H-33342 (blue). For quantitative assessment of total neurite mass, cells were analysed by an automated microscope system with imaging algorithms allowing a separation between cell bodies and cell extensions.

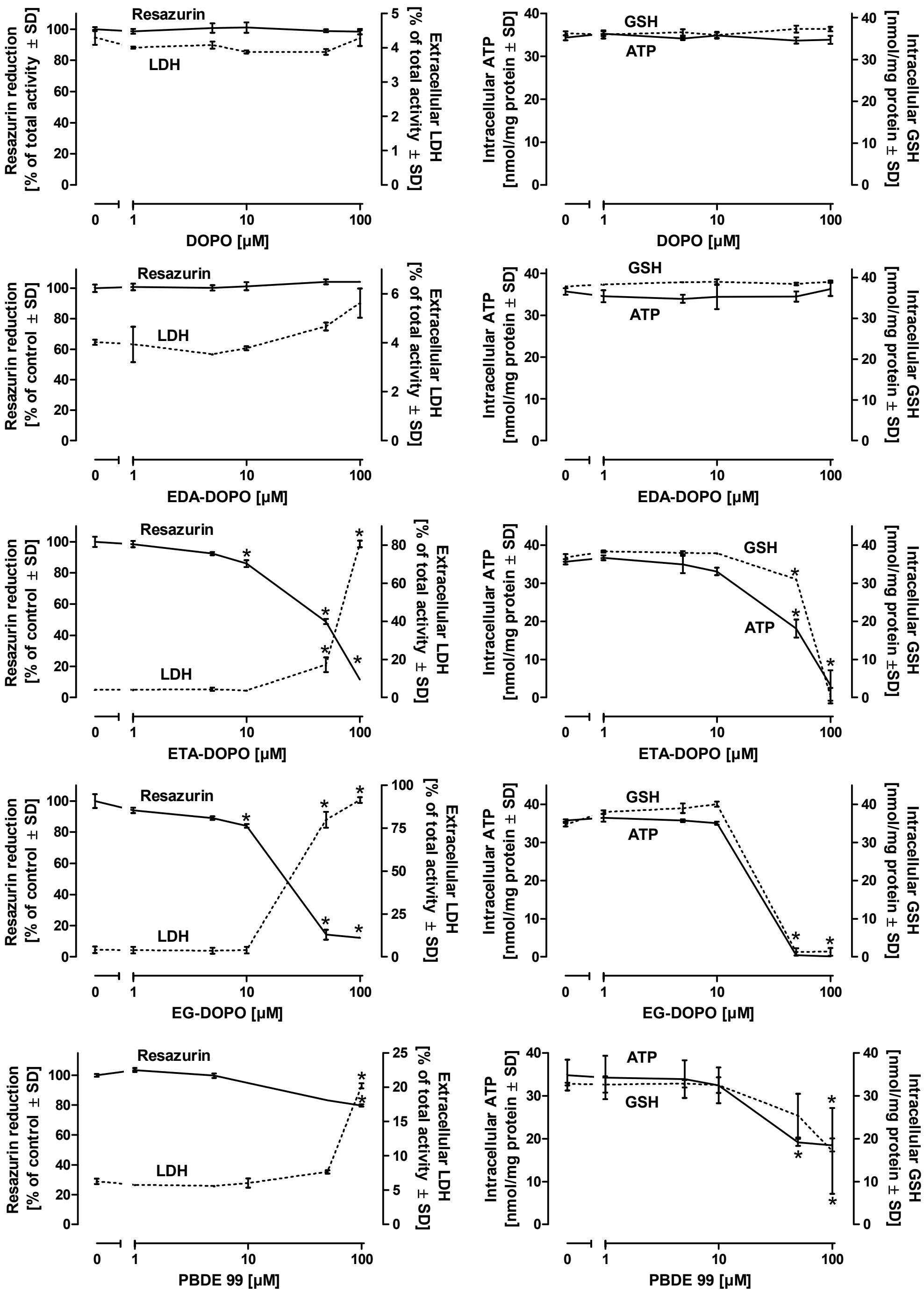

Suppl. Fig. 9. Toxicity of flame retardants in central human neurons. Differentiating human dopaminergic neurons (LUHMES) were treated with the different flame retardants in the concentrations as indicated for 48 h from day 0 to day 2 of differentiation. **a** Cell viability was determined by the resazurin reduction and by the lactate dehydrogenase release assays. **b** Intracellular levels of ATP and of reduced glutathione (GSH) were measured in parallel.

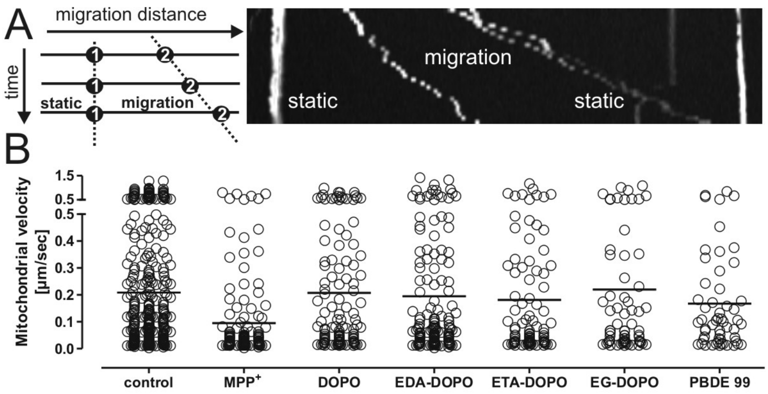

Suppl. Fig. 10. Migration of mitochondria in neurites. Mitochondrial migration was detected in fully differentiated (day 6 of differentiation, DoD6) mixed LUHMES cultures consisting of 3% eGFP/mito-tRFP-overexpressing LUHMES in 97% unlabeled LUHMES in order to allow tracking of mitochondria in single neurites in an environment of normal LUHMES cell density (Schildknecht et al. 2013, ALTEX 30(4):427-444). Single cell time lapse imaging was performed for a period of 90 s in intervals of 3 s with a Zeiss LSM 780 inverted fluorescence microscope YOKOGAWA CSU-XI spinning disk system using an EVOLVE camera (Carl Zeiss Group, Oberkochen, Germany) equipped with a 5% CO_2 /37°C controlled chamber and a 40 x 1.3 NA oil objective. For image analysis, kymographs (graphical representation of spatial position over time) based on the data obtained from single cell time lapse experiments were created using ImageJ Software (NIH, Bethesda, USA). The neurite was manually defined by the segmented line tool. To calculate mitochondrial velocity, diagonal lines were drawn for each moving mitochondrium in the kymograph and analysed by calculating the slope with the macro-plugin Kymo Line ROI, generated by Felix Schöneberger, Bioimaging Center (BIC), University of Konstanz, Germany.

Synthesis of DOPO-derivatives

9,10-dihydro-9-oxa-10-phosphaphenanthrene-10-oxide (DOPO) was purchased from Metadynea GmbH (Austria), all other chemicals were purchased from Sigma Aldrich (Switzerland) and were used without further purification. All ^{1}H, ^{13}C and ^{31}P NMR spectra were recorded. NMR spectra were collected at 298°K ambient temperature on a Bruker Avance III 400 NMR spectrometer (Bruker Biospin AG, Fällanden, Switzerland) at 400.2, 100.6 and 162.0 MHz, respectively, using a Bruker Avance 400 NMR spectrometer. ^{1}H and ^{13}C chemical shifts (δ) in ppm are calibrated to residual solvent peaks (DMSO-d6: δ = 2.49 and 39.5 ppm), the ^{31}P chemical shifts were referenced to an external sample with neat H_3PO_4 at 0.0 ppm. ^{1}H and $^{13}C\{H\}$ NMR spectra were referenced against the solvent (DMSO-d6). $^{31}P\{H\}$ NMR spectra were referenced against external 85% H_3PO_4. Mixture of diastereomers was found. For EG-DOPO, ETA-DOPO and EDA-DOPO, disabling the discrimination of the generally doubled set of ^{13}C signals of the individual species. EDA-DOPA and ETA-DOPO were synthesized according to relevant literature procedure with slight modifications (Gaan et al. 2013; Buczko et al. 2014). EG-DOPO was synthesized as previously reported with modifications (Kobayashi et al. 2013)

Synthesis of 6,6'-(ethane-1,2-diylbis(oxy))bis(6*H*-dibenzo[*c,e*][1,2]oxaphosphinine-6-oxide) (EG-DOPO): A four neck round bottom flask (1 L) connected to additional funnel, condenser, mechanical stirrer, thermometer and bubbler, was charged with DOPO (100 g, 0.46 mol), Ethylene glycol (14.35 g, 0.23 mol), 1-methylimidazole (39.88 g, 0.48 mol) and CH_3CN (300 mL). The reaction vessel was immersed in an ice bath and CCl_4 (72.93 g, 0.47 mol) was added under N_2 at a rate such that the reaction temperature did not exceed 10°C. After complete addition, the reaction temperature was allowed to warm up to ambient temperature, and the reaction was refluxed for 3 h. The reaction was then cooled down to 40°C, and poured into DCM (300 mL). The organic was then washed with water (3 x 500 mL) and brine (1 x 500 mL) and dried over Na_2SO_4. The organic solution was filtered and the volatiles were removed under reduced pressure. The solid was then dissolved in minimum amount of Ethanol. Water (4 L) was then added slowly with vigorous stirring. The mixture was then stirred overnight. The aqueous solution was decanted and the washing step was repeated until the product becomes hard solid. The product was collected and extra dried under vacuum at 60°C. Yield: 72.5 g, (64%), m.p. 110.4°C and 147.6°C. ^{1}H NMR (DMSO-d6, 400.2 MHz) δ (ppm): 8.23-8.20 (m, 2H), 8.17-8,14 (m, 2H), 7.84-7.80 (m, 2H), 7.76-7.68 (m, 2H), 7.57-7.52 (m, 2H), 7.45-7.40 (m, 2H), 7.34-7.30 (m, 2H), 7.21-7,19 (m, 2H), 4.28-4.08 (m, 4H). ^{13}C NMR (DMSO-d6, 100.6 MHz) δ (ppm): 149.09, 149.01, 136.24, 136.22, 136.17, 136.15, 134.03, 134.01, 130.88, 129.78, 129.68, 128.75, 128.60, 125.82, 125.07, 124.68, 124.56, 122.26, 121.86, 121.74, 120.47, 119.88, 119.81, 65.36, 65.34, 65.30, 65.28, 65.24, 65.22. ^{31}P NMR (DMSO-d6, 162.0 MHz) δ (ppm) 9.91A four neck round bottom flask (1 L) connected to additional funnel, condenser, mechanical stirrer, thermometer and bubbler, was charged with DOPO (100 g, 0.46 mol), Ethylene glycol (14.35 g, 0.23 mol), 1-methylimidazole (39.88 g, 0.48 mol) and CH_3CN (300 mL). The reaction vessel was immersed in an ice bath and CCl4 (72.93 g, 0.47 mol) was added under N_2 at a rate such that the reaction temperature did not exceed 10°C. After complete addition, the reaction temperature was allowed to warm up to ambient temperature, and the reaction was refluxed for 3 h. The reaction was then cooled down to 40°C, and poured into DCM (300 ml). The organic was then washed with water (3 x 500 ml) and brine (1 x 500 ml) and dried over Na_2SO_4. The organic solution was filtered and the volatiles were removed under reduced pressure. The solid was then dissolved in minimum amount of Ethanol. Water (4 L) was then added slowly with vigorous stirring. The mixture was then stirred overnight. The aqueous

solution was decanted and the washing step was repeated until the product becomes hard solid. The product was collected and extra dried under vacuum at 60°C. Yield: 72.5 g, (64 %), m.p. 110.4°C and 147.6°C. Set of diastereomers: ^{1}H NMR (DMSO-d6, 400.18 MHz) δ (ppm): 8.23-8.20 (m, 2H), 8.15 (dd, 2H, J = 8.0 Hz, J = 1.5 Hz), 7.84-7.80 (m, 2H), 7.75-7.68 (m, 2H), 7.54 (ds, 2H, J = 3.7 Hz, J = 0.8 Hz), 7.45-7.40 (m, 2H), 7.34-7.30 (m, 2H), 7.20 (dd, 1H, J = 2.7 Hz, J = 1.2 Hz), 7.18 (dd, 1H, J= 2.7 Hz, J= 1.2 Hz), 4.28-4.08 (m, 4H). ^{13}C NMR (DMSO-d6, 100.62 MHz) δ (ppm): 149.05 (d, J = 7.7 Hz), 136.19 (d, J = 7.2 Hz), 134.02 (d, J = 2.5 Hz), 130.88, 129.73 (d, J = 9.3 Hz), 128.67 (d, J = 15.2 Hz), 125.82, 125.07, 124.62 (d, J = 11.8 Hz), 121.80 (d, J = 11.8 Hz), 121.36 (d, J = 179.3 Hz), 119.84 (d, J = 6.6 Hz), 65.29 (dt, J = 5.9 Hz, J = 1.9 Hz). ^{31}P NMR (DMSO-d6, 161.99 MHz) δ (ppm) 9.91

Synthesis of 6,6'-(ethane-1,2-diylbis(azanediyl))bis(6*H*-dibenzo[*c,e*][1,2]oxaphosphinine-6-oxide) (EDA-DOPO): A four neck round bottom flask (1 L) connected to additional funnel, condenser, mechanical stirrer, thermometer and bubbler, was charged with DOPO (75 g, 0.35 mol), Ethylenediamine (8.34 g, 0.14 mol), Triethylamine (38.62 g, 0.38 mol) and $CHCl_3$ (400 mL). The reaction vessel was immersed in an ice bath, and CCl_4 (58.70 g, 0.38 mol) was added under N_2 at a rate such that the reaction temperature did not exceed 10°C. After complete addition, the reaction temperature was allowed to warm up to ambient temperature and stirred overnight. The white product was then collected by filtration and was washed with $CHCl_3$, Ethanol and then water, respectively. The product was collected and extra dried under vacuum at 60°C. Yield: 60.1 g, (88.7%), m.p. 255-257°C. ^{1}H NMR (400.2 MHz, DMSO-d6) δ (ppm): 8.17- 8.11 (m, 4H), 7.80-7.76 (m, 1H), 7.75-7.70 (m, 3 H), 7.56-7.49 (m, 2H), 7.43-7.38 (m, 2H), 7.30-7.25 (m, 2H), 7.18-7.13 (m, 2H), 5.76-5.71 (m, 2H), 2.89-2.81 (m, 4H). ^{13}C NMR (100.6 MHz, DMSO-d6) δ (ppm): 149.44, 149.41, 149.37, 149.34, 141.70, 136.00, 135.99, 135.93, 135.92, 134.23, 132.74, 132.73, 132.72, 130.38, 130.36, 129.49, 129.46, 129.39, 129.36, 128.44, 128.41, 128.30, 128.27, 126.04, 126.03, 125.43, 125.42, 125.40, 125.39, 124.43, 124.28, 124.16, 124.13, 124.06, 124.02, 121.98, 121.87, 120.10, 120.05, 120.04, 120.00, 41.77, 41.72. ^{31}P NMR (162.0 MHz, DMSO-d6) δ (ppm): 15.29, 15.25.A four neck round bottom flask (1 L) connected to additional funnel, condenser, mechanical stirrer, thermometer and bubbler, was charged with DOPO (75 g, 0.35 mol), Ethylenediamine (8.34 g, 0.14 mol), Triethylamine (38.62 g, 0.38 mol) and $CHCl_3$ (400 mL). The reaction vessel was immersed in an ice bath, and CCl_4 (58.70 g, 0.38 mol) was added under N_2 at a rate such that the reaction temperature did not exceed 10°C. After complete addition, the reaction temperature was allowed to warm up to ambient temperature and stirred overnight. The white product was then collected by filtration and was washed with $CHCl_3$, Ethanol and then water, respectively. The product was collected and extra dried under vacuum at 60°C. Yield: 60.1 g, (88.7%), m.p. 255-257°C. Set of diastereomers: ^{1}H NMR (400.18 MHz, DMSO-d6) δ (ppm): 8.17- 8.11 (m, 4H), 7.80-7.76 (m, 1H), 7.75-7.70 (m, 3 H), 7.56-7.49 (m, 2H), 7.43-7.38 (m,2H), 7.30-7.25 (m, 2H), 7.18-7.13 (m, 2H), 5.76-5.71 (m, 2H), 2.89-2.81 (m, 4H). ^{13}C NMR (100.62 MHz, DMSO-d6) δ (ppm): 149.41 (d, J = 7.2 Hz), 135.97 (d, J = 6.8 Hz), 132.74, 130.37, 129.41 (d, J = 9.6 Hz), 128.34 (d, J = 14.3 Hz), 125.42 (d, J = 0.8 Hz), 125.24 (d, J = 161.7 Hz), 124.27, 124.08 (d, J = 10.7 Hz), 121.92 (d, J = 11.5 Hz), 120.05 (d, J = 5.8 Hz), 41.75 (d, J = 5.7 Hz). ^{31}P NMR (161.99 MHz, DMSO-d6) δ (ppm): 15.29, 15.25.

Synthesis of 6-(2-((6-oxido-6*H*-dibenzo[*c,e*][1,2]oxaphosphinin-6-yl)amino)ethoxy)-6*H*-dibenzo[*c,e*][1,2]oxaphosphinine-6-oxide) (ETA-DOPO): A four neck round bottom flask (1 L) connected to additional funnel, condenser, mechanical stirrer, thermometer and bubbler, was charged with DOPO (150 g, 0.63 mol) , Ethanolamine (21.2 g, 0.34 mol), Triethylamine (77.23 g, 0.76 mol) and $CHCl_3$ (400 mL). The reaction vessel was immersed in an ice bath, and CCl_4 (117.40 g, 0.76 mol) was added under N_2 at a rate such that the reaction temperature did not exceed 10°C. After complete addition, the reaction temperature was allowed to warm up to ambient temperature and stirred overnight. The organic solvent was then concentrated under reduced pressure and the white precipitate was collected by filtration. The filtrate was then completely evaporated under reduced pressure. The white product was then combined and washed with ethanol and water successively. The product was collected and extra dried under vacuum at 60°C. Yield: 135.8 g, (80%), m.p. 177-178°C. Mixture of diastereomers with ratio of 1:1, ^{1}H NMR (400.2 MHz, DMSO-d6) δ (ppm): 8.27-8.23 (m, 1H), 8.21-8.13 (m, 3H), 7.93-7.83 (m, 2H), 7.74-7.69 (m, 1H), 7.68-7.59 (m, 2H), 7.48-7.39 (m, 3H), 7.36-7.32 (m,1H), 7.31-7.26 (m, 2H), 7.18-7.12 (m, 1H), 5.87-5.80 (m, 1H), 4.13-3.94 (m, 2H), 3.11-2.91 (m, 2H). ^{13}C NMR (100.6 MHz, DMSO-d6) δ (ppm): 149.35, 149.34, 149.28, 149.27, 149.24, 149.23, 149.16, 149.15, 136.29, 136.28, 136.22, 136.20, 135.96, 135.93, 135.89, 135.87, 134.04, 134.01, 132.76, 132.74, 130.93, 130.92, 130.40, 129.98, 129.96, 129.89, 129.87, 129.48, 129.38, 128.84, 128.83, 128.69, 128.68, 128.36, 128.33, 128.22, 128.19, 126.11, 126.09, 125.90, 125.88, 125.87, 125.41, 125.09, 124.72, 124.61, 124.49, 124.47, 124.30, 124.13, 124.11, 124.02, 124.00, 122.45, 122.02, 122.00, 121.97, 121.96, 121.90, 121.88, 121.85, 121.84, 120.68, 120.14, 120.11, 120.08, 120.05, 119.92, 119.90, 119.89, 119.83, 66.19, 66.16, 66.14, 66.13, 66.11, 66.10, 66.07, 66.05, 66.04. ^{31}P NMR (162.0 MHz, DMSO-d6) δ (ppm): 14.59, 14.57, 9.83, 9.79 A four neck round bottom flask (1 L) connected to additional funnel, condenser, mechanical stirrer, thermometer and bubbler, was charged with DOPO (150 g, 0.63 mol) , Ethanolamine (21.2 g, 0.34 mol), Triethylamine (77.23 g, 0.76 mol) and $CHCl_3$ (400 mL). The reaction vessel was immersed in an ice bath, and CCl_4 (117.40 g, 0.76 mol) was added under N_2 at a rate such that the reaction temperature did not exceed 10°C. After complete addition, the reaction temperature was allowed to warm up to ambient temperature and stirred overnight. The organic solvent was then concentrated under reduced pressure and the white precipitate was collected by filtration. The filtrate was then completely evaporated under reduced pressure. The white product was then combined and washed with ethanol and water successively. The product was collected and extra dried under vacuum at 60°C. Yield: 135.8 g, (80%), m.p. 177-178°C. Set of diastereomers: ^{1}H NMR (400.18 MHz, DMSO-d6) δ (ppm): 8.25 (t, J = 7.2 Hz, J = 1H), 8.21-8.12 (m, 3H), 7.93-7.83 (m, 2H), 7.74-7.69 (m, 1H), 7.68-7.59 (m, 2H), 7.48-7.39 (m, 3H), 7.34 (dt, J = 0.8 Hz, J = 7.6 Hz, 1H), 7.31-7.26 (m, 2H), 7.15 (ddd, J = 1.0 Hz, J = 8.1 Hz, J = 14.0 Hz, 1H), 5.87-5.80 (m, 1H), 4.13-3.94 (m, 2H), 3.11-2.91 (m, 2H). ^{13}C NMR (100.62 MHz, DMSO-d6) δ (ppm): 149.35-149.15 (8 resonances), 136.29-135.87 (8 resonances), 134.03 (d, J = 2.4 Hz), 132.75 (d, J = 2.2 Hz), 130.92 (d, J = 0.8 Hz), 130.40), 129.93 (dd, J = 2.0 Hz, J = 9.3 Hz), 129.43 (d, J = 9.6 Hz), 128.75 (dd, J = 1.1 Hz, J = 15.1 Hz), 128.27 (dd, J = 3.2 Hz, J = 14.3 Hz), 125.90-125.87 (3 resonances), 125.41, 125.29 (dd, J = 2 Hz, J = 163.0 Hz), 125.09, 124.66 (d, J =11.7 Hz), 124.30, 124.06 (dd, J = 1.3 Hz, J = 11.1 Hz), 122.02-121.84 (8 resonances), 121.57 (d, 178.8 Hz), 120.14-120.05 (4 resonances), 119.92-119.83 (4 resonances), 66.19-66.03 (m), 40.43 (d, J = 7.0 Hz). ^{31}P NMR (161.99 MHz, DMSO-d6) δ (ppm): 14.59, 14.57, 9.83, 9. 79

References

Buczko A, Stelzig T, Bommer L, rentsch D, Heneczkowski M, Gaan S (2014) Bridged DOPO derivatives as flame retardants for PA6. Poly Degrad Stab 107:158-165

Gaan S, Neisius M, Mercoli P, Liang S, Mispreuve H, Naescher R (2013) Phosphonamidates, production methods and flame retardant applications. WO2013020696 A2

Kobayashi J, Ishikawa A, Miwa K, Iguchi S (2013) Flame retarder comprising condensed phosphonic acid ester and flame-retardant resin composition. Patent WO 2013015376
